# Assessing acute thermal assays as a rapid screening tool for coral restoration

**DOI:** 10.1038/s41598-024-51944-5

**Published:** 2024-01-22

**Authors:** C. N. Klepac, C. G. Petrik, E. Karabelas, J. Owens, E. R. Hall, E. M. Muller

**Affiliations:** 1 Mote Marine Laboratory, International Center for Coral Reef Research and Restoration, Summerland Key, FL USA; 2 Present Address: Hopkins Marine Station, Stanford University, Pacific Grove, CA USA; 3 National Coral Reef Institute, Nova Southeastern University, Dania Beach, FL USA; 4 Tufts University, Worcester, MA USA; 5 Mote Marine Laboratory, Sarasota, FL USA

**Keywords:** Marine biology, Physiology

## Abstract

Escalating environmental threats to coral reefs coincides with global advancements in coral restoration programs. To improve long-term efficacy, practitioners must consider incorporating genotypes resilient to ocean warming and disease while maintaining genetic diversity. Identifying such genotypes typically occurs under long-term exposures that mimic natural stressors, but these experiments can be time-consuming, costly, and introduce tank effects, hindering scalability for hundreds of nursery genotypes used for outplanting. Here, we evaluated the efficacy of the acute Coral Bleaching Automated Stress System (CBASS) against long-term exposures on the bleaching response of *Acropora cervicornis*, the dominant restoration species in Florida’s Coral Reef. Comparing bleaching metrics, *F*_v_/*F*_m_, chlorophyll, and host protein, we observed similar responses between the long-term heat and the CBASS treatment of 34.3 °C, which was also the calculated bleaching threshold. This suggests the potential of CBASS as a rapid screening tool, with 90% of restoration genotypes exhibiting similar bleaching tolerances. However, variations in acute bleaching phenotypes arose from measurement timing and experiment heat accumulation, cautioning against generalizations solely based on metrics like *F*_v_/*F*_m_. These findings identify the need to better refine the tools necessary to quickly and effectively screen coral restoration genotypes and determine their relative tolerance for restoration interventions.

## Introduction

Chronic ocean warming and associated increases in the frequency and severity of marine heat waves are resulting in near-annual mass coral bleaching events^1,2^, and subsequent widespread coral mortality. Despite drastic declines in coral cover globally, it is well documented that coral species, populations, and individuals possess inherent or acclamatory tolerance to increased thermal stress^3–8^. In order to mitigate the critical loss of ecosystem functioning, stress-tolerant corals need to be identified and protected concomitant with biological interventions aimed at maximizing coral growth, survival, and genetic diversity in degraded reef habitats.

In Florida’s Coral Reef (FCR), reef-building coral cover has been reduced to ~ 2% over the last 50 years^9^ from a combination of ocean warming, disease, and storms^9–11^, leaving most of these reef ecosystems functionally extinct. As a result, coral restoration programs have emerged with the aim to propagate and outplant tens of thousands of coral fragments annually, in an attempt to buffer continued reef degradation. However, restoration practitioners spend immense amounts of time, effort, and capital to put coral biomass on reef habitats^12^, despite continued and unabated global and local threats that result in reduced survival following as early as two years post-outplanting^13,14^. Some restoration nurseries contain hundreds of genotypes per species and these nursery populations are likely to contain a wide distribution of heat tolerant individuals^15^. Incorporating tolerant nursery corals into outplanting and assisted sexual breeding efforts has the potential to bolster the resilience of restored populations, as long as heat tolerance is durable and not maladaptive for disease resistance^16^, growth^17^ (but see^4,18–20^), or other life history traits. Biological interventions using tolerant corals can increase the likelihood of ecological buffering and long-term persistence on otherwise degraded reefs.

Investigating nursery corals' relative resistance or susceptibility to climate change scenarios is critical to surmise potential outplant success following coral bleaching events. Wild coral populations demonstrate substantial variation in thermal tolerance across latitudes, environmental gradients, and coral species^3,15,21–24^. But even within relatively small spatial scales (< 1 km), individuals exhibit different bleaching responses^25–27^ attributed to symbiont composition^28^, microbial consortium^29^, and/or fixed genetic effects^18,30^. In a restoration nursery setting, individual corals have been common-gardened under similar environmental conditions for years and largely host the same algal symbiont type^31,32^, but still display considerable bleaching variation, suggesting adaptive capacity in bleaching traits^15,33,34^. Therefore, the proactive selection of corals with some level of climate resilience while maintaining genetic diversity is a promising intervention to enhance the adaptive capacity of threatened restoration nursery corals.

To date, much research quantifying restoration coral thermal tolerance occurs under long-term thermal exposures^20,33,34^. Exposures that mimic natural warming rates and bleaching phenotypes can be monitored closely to avoid spurious mortality, yet, these experimental systems are costly to run and maintain, are limited in individual replication, often require destroying fragments of critically endangered coral species, and tank acclimation effects are possible^35^. Quantifying fine-scale differences among every nursery genotype (per species) in a long-term experimental setting is also a significant time investment. Consequently, many of these genotypes are being outplanted to degraded reef habitats without empirical evidence of their tolerance, which is a serious impediment to advancing restoration success in the long term. Recent developments in rapid quantification of coral thermal tolerance using the low-cost, portable Coral Bleaching Automated Stress System^36–38^ (CBASS) have demonstrated similar coral bleaching responses between chronic and acute heat stress assays, as well as resolving population-level differences exhibited following natural bleaching^25,36,38–40^ although genotype differences or rankings within populations remains equivocal. Moreover, a comparative study investigating coral stress responses under acute and chronic thermal stress found an overlap in holobiont performance. Additionally, the algal metric *F*_v_/*F*_m_ was used to quantify upper thermal thresholds of corals, termed *F*_v_/*F*_m_ ED50^37,38^, or the temperatures at which *F*_v_/*F*_m_ declines by 50% relative to baseline values. While ED50 is not an absolute measure of thermal tolerance, it has demonstrated a potential utility in capturing a broad census of thermal tolerances in wild^4,23^ and nursery^15^ populations.

**Figure 1:**
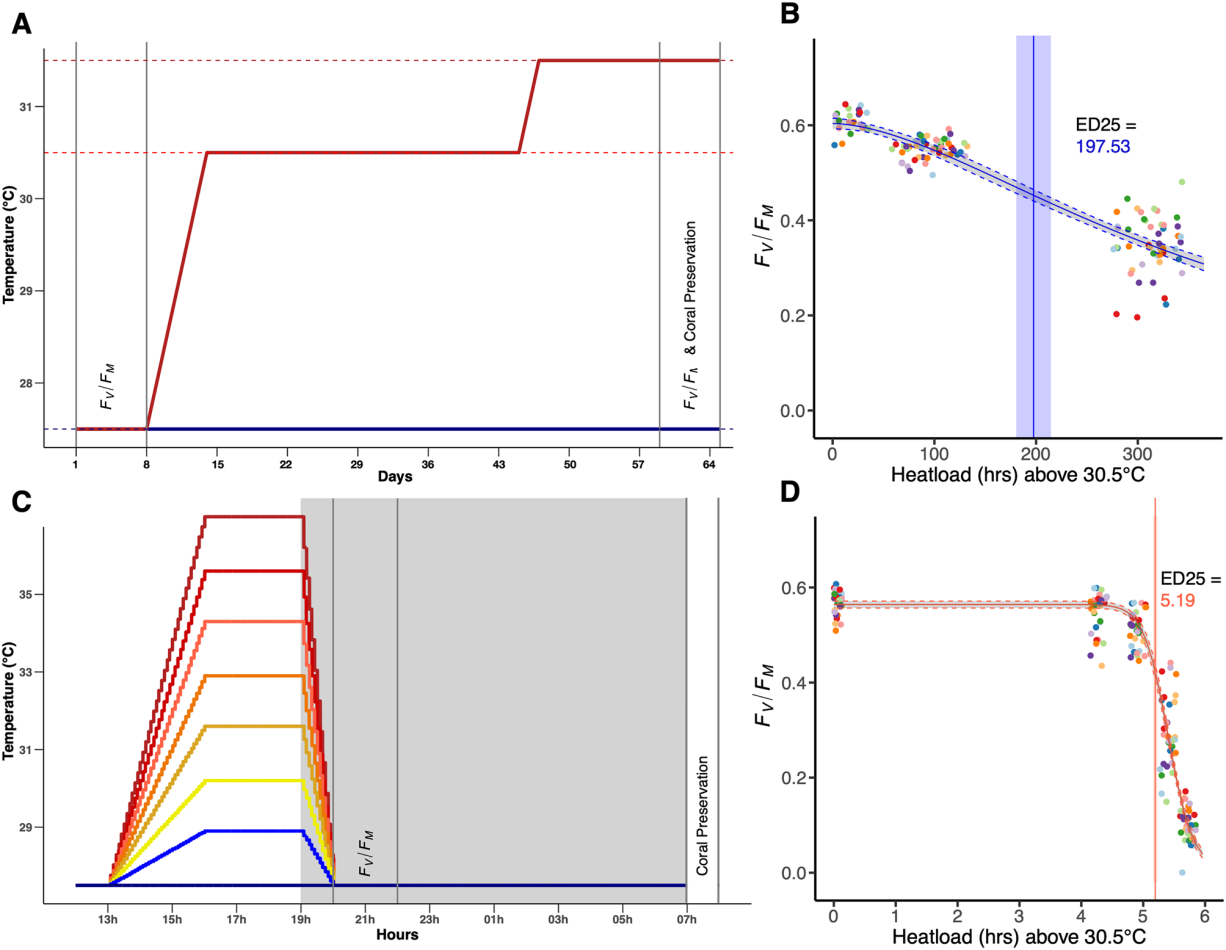
Experimental design for comparing coral bleaching responses between chronic and acute thermal stress. (A&C) Thermal profiles and sampling time points for physiological measurements, ramping up from ambient mean annual temperature (27.5 °C) to targeted experiment treatment temperatures. After 8 days of acclimation, the long-term (LT; A) control (blue line) and heat (OW; red line) commenced for 2 months. The 18-h CBASS (C) was run in 8 independently-controlled temperature tanks, where each coral genotype was exposed to each temperature treatment, replicated across three rounds (CBASS) and five tanks (LT). (B&D) Dose–response curves modeling *F*_v_/*F*_m_ as a function of the number of hours over the regional bleaching threshold of 30.5 °C to estimate ED25 (blue [LT; B] and red [CBASS; D] vertical lines) for each experiment.

To examine the efficacy of replacing long-term experiments with the acute CBASS experiment in a restoration context, we compared the bleaching responses of ten genotypes of *Acropora cervicornis,* a subset of hundreds of genets currently within propagation, under a long-term (LT) two-month bleaching exposure and acute CBASS (Fig. 1A) exposures at Mote Marine Laboratory’s International Center for Coral Reef Research and Restoration (MML-IC2R3). Threatened *A. cervicornis* has been the primary focus of restoration programs along FCR, due to its fast growth, morphology, and life-history strategy^41^ and is the most outplanted coral species in the Atlantic and Caribbean region. Yet, Acroporids are typically recognized as heat-intolerant genera^42^ and Caribbean *A. cervicornis* hosts the thermally sensitive symbiont (*Symbiodinium fitti*^43–45^). Thus, it was imperative to quantify bleaching thresholds on a subset of this nursery population to determine whether this approach is scalable for actionable and successful restoration strategies. We discuss similarities and differences between the two types of heat exposure, highlighting experimental considerations, how phenotypic traits respond under different rates and the value, as well as challenges, of acute heat stress assays in measuring coral thermal tolerance in a restoration context.

## Results

### Detecting bleaching phenotypes between long-term and acute thermal exposures

Previous research incorporating CBASS experiments utilizes the non-invasive metric of *F*_v_/*F*_m_ to characterize bleaching across multiple temperatures and calculate bleaching thresholds for coral species and/or populations^15,36–38^. *F*_v_/*F*_m_ values fitted to a log-logistic regression dose–response curve are used to derive Effective Dose values, such as ED25 and ED50, to attribute 25% or 50% of the thermal bleaching threshold, respectively. ‘Heatloding ED25,’ or the amount of time in hours over the regional bleaching threshold (30.5 °C) to reach a 25% *F*_v_/*F*_m_ reduction in heated coral replicates, was calculated to compare bleaching detection between two experiments of different lengths^46^. Within the present study, detecting a 25% bleaching phenotype in ten nursery genotypes of *Acropora cervicornis* took longer under the long-term (LT; 2-month) than the acute (18-h) experiment (Fig. 1B). It took 197.53 h, or 8.2 days, in the LT ocean warming conditions (LT-OW) treatment whereas it took only 5.19 h to detect 25% bleaching in CBASS replicates.

**Figure 2:**
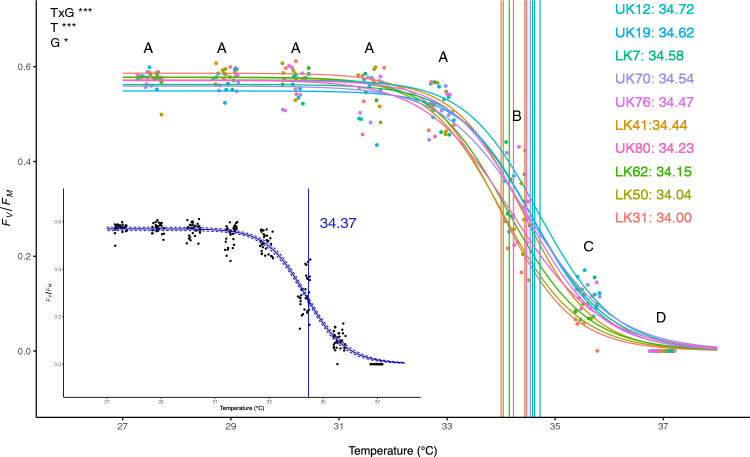
Maximum quantum yield (*F*_v_/*F*_m_) of nursery *A. cervicornis* genotypes (n = 10) at the end of the CBASS heating ramp, fitted to a log-logistic dose–response curve. ED50 metrics for the entire nursery population (inset blue vertical line) and each genotype (colored vertical lines), ordered by highest to lowest ED50. Capital letters denote differences among CBASS treatments. Significant main and interactive effects of Treatment (T) and Genotype (G) are reported (*p < 0.05, **p < 0.01, ***p < 0.0001).

In addition to deriving the amount of heat accumulation to detect bleaching, bleaching thresholds (ED50) were quantified for CBASS samples by fitting *F*_v_/*F*_m_ values to a dose–response curve as a function of maximum treatment temperature. Under CBASS, the calculated ED50 bleaching threshold value for MML’s nursery population of *A. cervicornis* was 34.37 °C (Fig. 2). Among genotypes, variation in ED50 values was only 0.7 °C, ranging from 34.0 to 34.7 °C (± 0.2 S.D.), where four of the top five ED50 thresholds were from genotypes originally sourced from the Upper Florida Keys (Table S1). The top Upper Keys genotypes were UK12 (34.7 ± 0.2) and UK19 (34.6 ± 0.2), followed by Lower Keys genotype LK7 (34.6 ± 0.2). To compare the effects of coral source region on ED50 values, we conducted an independent t-test and found no significant difference in ED50 values (*p* = 0.088) among Upper and Lower Keys corals. For the LT experiment, ED50 thresholds of nursery genotypes could not be calculated since a 50% reduction in *F*_v_/*F*_m_ values did not occur for all genotypes, a criterion needed to fit 3-parameter log-logistic curves.

### Comparison of coral response traits between long-term and acute bleaching treatments

**Figure 3:**
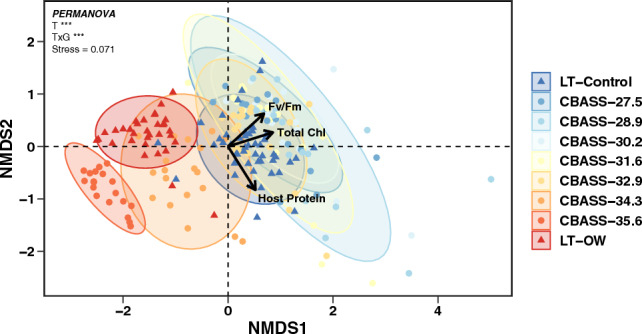
Non-metric multidimensional scaling (NMDS) plot of coral phenotypic trait similarities within temperature treatments of the LT (triangles) and CBASS (circles) experiments. Trait vectors depict directional differences between treatments determined by PERMANOVA, with the length of each vector displaying the strength of each trait. Ellipses represent 95% confidence intervals. The CBASS 37 °C treatment was removed due to high coral mortality during the recovery period. Significant main and interactive effects of Treatment (T) and Genotype (G) are reported (*p < 0.05, **p < 0.01, ***p < 0.0001).

To determine which CBASS treatment(s) yielded comparable bleaching responses to the LT experiment, *F*_v_/*F*_m_, total chlorophyll, and host soluble protein were examined among all experimental treatments. There was substantial overlap in multivariate phenotypic traits in response to heat stress between the LT and CBASS-34.3 °C treatment (PERMANOVA *p*.adjust = 1; Fig. 3; Table S7), or the derived bleaching threshold. The remaining CBASS treatments had different physiological responses from the LT-OW treatment (*p*.adjust = 0.035) but were not different from CBASS 34.3 °C. All response variables contributed similarly to the ordination of experimental treatments (*F*_v_/*F*_m_ R^2^ = 0.862, total chlorophyll R^2^ = 0.803, host protein R^2^ = 0.984). Interestingly, all CBASS treatments and LT-OW were similar to the LT-Control treatment (*p*.adust = 1). Upon further exploration within the LT experiment, there was an effect of tank photoacclimation during the two-month exposure, where LT-Control initial *F*_v_/*F*_m_ values were on average 1.5-fold higher than final *F*_v_/*F*_m_ values (Treatment*Time *p* < 0.0001; Fig. S1). Therefore, *F*_v_/*F*_m_ values for both experiment types were calculated as relative change (LT: [*F*_v_/*F*_mFinal_–*F*_v_/*F*_mInitial_]_Heat_–[*F*_v_/*F*_mFinal_–*F*_v_/*F*_mInitial_]_Control_; CBASS: *F*_v_/*F*_mHeat_-*F*_v_/*F*_m27.5 °C_) for subsequent analyses in order to account for the reduction in *F*_v_/*F*_m_ in LT-Control corals.

**Figure 4:**
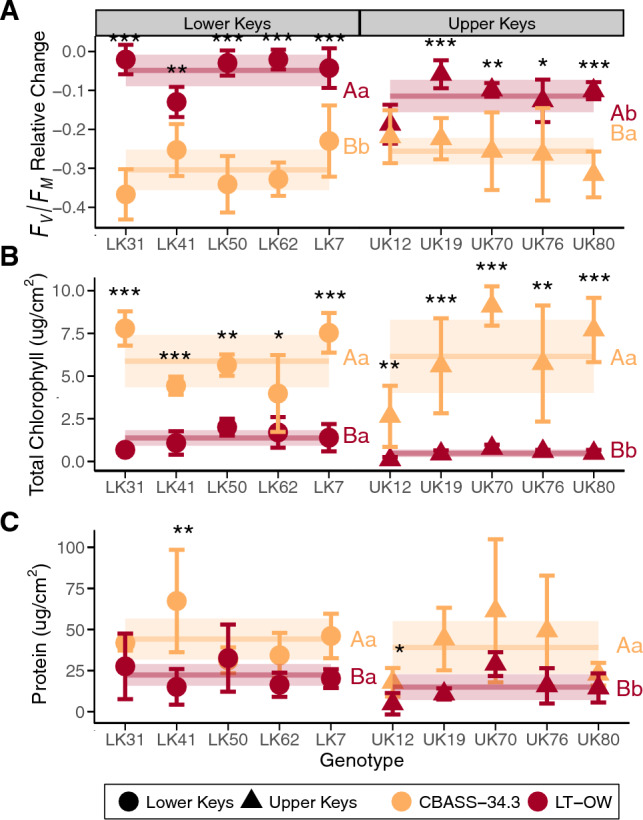
Phenotypic traits of (A) the relative change in *F*_v_/*F*_m_, (B) total chlorophyll (µg cm^-2^), and (C) host soluble protein (µg cm^-2^) by genotype between the LT heat (LT-OW; red) and CBASS bleaching treatment of 34.3 °C (orange). Mean (± 95% CI) values for each genotype (n = 2–5 per treatment) are separated by coral source region: Lower Keys (circles) and Upper Keys (triangles), where solid horizontal lines represent mean population values with shaded 95% CI. Asterisks represent differences between experiments within each genotype (*p < 0.05, **p < 0.01, ***p < 0.0001), uppercase letters represent pairwise differences between experiments within coral source region, and lowercase letters represent differences between region within experiment.

Each phenotypic trait was then examined separately to offer a more fine-scale comparison between LT-OW and CBASS 34.3 °C. The relative change in *F*_v_/*F*_m_ was greater under the CBASS 34.3 °C treatment than under LT-OW (Type III ANOVA Exp *p* = 0.0001), with an average (± S.D.) relative change in *F*_v_/*F*_m_ of -0.277 ± 0.077 and -0.081 ± 0.067, respectively. For 90% of genotypes, there was a genotype by experiment interaction (ExG *p* < 0.0001; Fig. 4A). Similar to *F*_v_/*F*_m_, square-root transformed total chlorophyll values were different between the two experimental exposures (Type III ANOVA Exp *p* = 0.001) but LT-OW chlorophyll (0.936 ± 0.441 µg/cm^2^) was lower than CBASS 34.3 °C chlorophyll (6.008 ± 1.465 µg/cm^2^). CBASS 34.3 °C mean total chlorophyll values were sixfold greater than LT-OW total chlorophyll values, and as a result, every genotype comparison between the two experiments was significantly different (ExG *p* < 0.001, Fig. 4B). Host soluble protein (µg/cm^2^) was also greater under CBASS 34.3 °C (40.245 ± 16.980 µg/cm^2^; Welch’s *p* < 0.0001) than under LT-OW (18.523 ± 11.155 µg/cm^2^). Despite a qualitative difference in protein values among the LT-OW and CBASS 34.3 °C treatments, 80% of genotypes had similar protein values between experiments (ExG *p* = 0.5633; Fig. 4C).

### Genetic variation in coral bleaching response traits depends on the experiment type and source region

A goal of many restoration practitioners is to determine which individual genotypes from nursery broodstock are more resistant to climate change stressors^33^. Therefore, genotypic differences among and within both CBASS and LT bleaching treatments were compared for each trait. For the relative change in *F*_v_/*F*_m_ among experiments, there was a significant effect of genotype (G *p* < 0.05), but pairwise comparisons revealed a marginal difference only between LK7 and UK80 (*emmeans* *p* = 0.056). Within the bleaching CBASS treatment of 34.3 °C, genotypes UK12, UK19, and LK7 had a smaller relative change in *F*_v_/*F*_m_ than LK31 and LK50, and LK7 and UK76 had smaller reductions than LK31 (ExG *p* < 0.0001; Fig. 4A, Fig. S2B; Table S4). In contrast to the CBASS 34.3 °C treatment, UK12 had greater reductions in *F*_v_/*F*_m_ under LT-OW than genotypes LK31, LK50, LK62, LK7, and UK19, LK31, LK50, and LK62 had smaller relative change than LK41, and LK31 and LK62 had a smaller relative change in *F*_v_/*F*_m_ than UK76 (Fig. 4A, Fig. S2A; Table S4).

For total chlorophyll measured at the end of both experiments, there was a significant effect of genotype where UK12 had lower chlorophyll values than all other genotypes (G *p* < 0.0001). Unlike *F*_v_/*F*_m_ where UK12 was a ‘top’ performer under CBASS, UK12 had lower total chlorophyll than seven genotypes (= LK41 and LK62) in the CBASS 34.3 °C treatment and eight genotypes (= UK19) in the LT-OW treatment (Fig. 4B). Within the CBASS 34.3 °C treatment, LK62 had the second lowest chlorophyll content, with values lower than genotypes LK31, LK7, UK70, and UK80, followed by LK41 which was lower than UK70 (ExG *p* < 0.001; Fig. S2B; Table S5). Under LT-OW, LK50 had higher chlorophyll values than genotypes UK19, UK76, and UK80 (Fig. S2A; Table S5).

Similar to chlorophyll, genotype UK12 had lower overall host protein content than genotypes LK31, LK41, LK50, LK7, UK70, and UK76 (G *p* = 0.003). There were no genotypic differences in host protein concentrations within the CBASS bleaching treatment of 34.3 °C (*p* = 0.563; Fig. 4C, Fig. S2B; Table S6). However, under LT-OW, genotype UK12 had lower host protein values than LK31, LK50, LK7, and UK70 (Fig. S2A; Table S6).

Genotypic comparisons indicated possible effects of coral source region, therefore, population-level differences between the Upper Florida Keys and Lower Keys were compared using linear mixed models. To compare the effects of coral source region on ED50 values, we conducted an independent t-test and found no significant difference in ED50 values (*p* = 0.088) among Upper and Lower Keys corals. For the LT experiment, bleaching thermal thresholds of nursery genotypes could not be calculated since a 50% reduction in *F*_v_/*F*_m_ values did not occur for all genotypes. Yet, when we examined ED25 values for both CBASS and LT, there was no significant difference between source region for each experiment type (t-test LT *p* = 0.86, CBASS *p* = 0.20).

Regional differences in the relative change in *F*_v_/*F*_m_ were detected within both experiments but in opposite directions (ExR *p* < 0.0001). Under the CBASS bleaching treatment of 34.3 °C, the relative change in *F*_v_/*F*_m_ was smaller in Upper Keys (UK) sourced corals in comparison to Lower Keys (LK), yet the UK corals had a greater loss in *F*_v_/*F*_m_ values than LK corals under the LT-OW treatment (Fig. 4A, lowercase letters; Table S8). Additionally, Lower and Upper Keys genotypes had an overall smaller reduction in *F*_v_/*F*_m_ under the LT-OW treatment in comparison to CBASS 34.3 °C (*emmeans* *p* < 0.01; Fig. 4A, uppercase letters).

For both total chlorophyll and host protein concentration, there was an effect of experiment (E *p* < 0.05; Fig. 4B,C, capital letters) and the interaction of experiment and region for LT-OW only (ExR *p* < 0.05; Fig. 4B,C, lowercase letters). We did not find a regional effect for these ‘stress-accumulation’ bleaching traits under CBASS, but Lower Keys corals had overall higher amounts of total chlorophyll than Upper Keys corals. Trait mean values were greater under CBASS 34.3 °C than LT-OW, and LT-OW LK corals had higher chlorophyll and protein concentrations than UK corals (*emmeans* LK-UK *p* < 0.001; Fig. 4D; Table S8).

### Non-invasive photophysiology during acute stress is a poor predictor of bleaching outcomes

**Figure 5:**
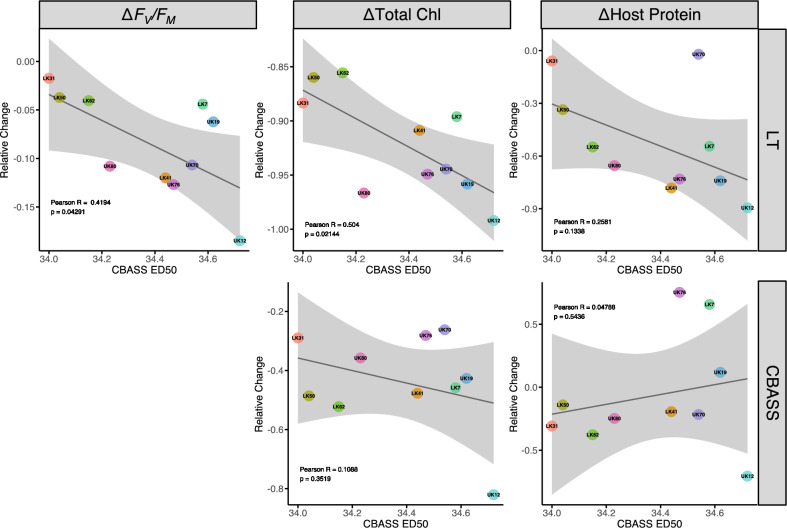
Correlations between each genotype’s ED50 value and the relative change in *F*_v_/*F*_m_, chlorophyll *a* (µg cm^-2^), and host protein (µg cm^-2^) for the LT-OW (top panel) and CBASS 34.3 °C (bottom panel) treatments. Trait relative change represents the difference between heat and control treatment, except for LT-OW *F*_v_/*F*_m_, which was final from initial values. Linear regression results are reported, and gray-shaded area represents 95% confidence intervals.

While considerable overlap for all coral responses between the LT-OW and CBASS 34.3 °C treatments was detected, there were large discrepancies among genotype comparisons for each trait. Therefore, using individual coral bleaching thresholds (CBASS ED50) we explored the relationship between each coral’s ED50 and both LT and CBASS bleaching responses. For all three traits – *F*_v_/*F*_m_, total chlorophyll, and host protein – each genotype's proportional change in trait values ((heat-control)/control) for LT-OW and CBASS 34.3 °C were regressed against the CBASS ED50 values (Fig. 5). Pearson correlations indicated there was a significant, but negative, relationship between ED50 and LT-OW *F*_v_/*F*_m_ and chlorophyll (*p* < 0.05). Regional effects detected in linear mixed models were only evident under LT-OW chlorophyll, where UK corals with greater ED50 values had the greatest loss in chlorophyll concentration. In all regressions, genotype UK12 which had the greatest ED50 value had the greatest relative loss in all traits under both experiment types; removal of this genotype did not significantly affect the resulting regressions.

Provided ED50 values were calculated from *F*_v_/*F*_m_ measurements taken during CBASS and LT *F*_v_/*F*_m_ measured at the end of the two-month exposure, we also explored the relationship between algal bleaching traits—*F*_v_/*F*_m_ and total chlorophyll. Although algal photophysiology can be characterized via different metrics, it could be assumed that *F*_v_/*F*_m_ values should be correlated with and used as a predictive proxy for bleaching outcomes measured by chlorophyll^47,48^. The loss in total chlorophyll was regressed against *F*_v_/*F*_m_ for both experiments, resulting in a strong positive relationship between *F*_v_/*F*_m_ and total chlorophyll under LT-OW (Pearson *p* < 0.001, R = 0.643; Fig. S3A). Under the CBASS 34.3 °C treatment however, there was no relationship between the two algal bleaching traits (Pearson *p* > 0.05, R = 0.116; Fig. S3B).

## Discussion

Along Florida’s Coral Reef, advances in the efficacy and scalability of nursery coral propagation and outplanting coupled with stress-testing have great potential to improve ecological restoration success and buffer degraded ecosystems. Yet, the most ecologically relevant method to assess species responses to climate change-associated stressors is long-term exposures (LT; 1–2 months) that are limited in scalability (number and replication of genotypes), efficiency (time and cost), and precision (potential for tank acclimation). Here, we compared the physiological responses to chronic (2 mo) and acute (18 h) thermal stress in the prioritized Caribbean restoration coral species *A. cervicornis*, to increase scalability and standardization of thermal resistance screening. An advantage of using CBASS over LT experiments was the ability to determine nursery population thermal thresholds^15^, which was 34.37 °C, and individual thresholds, which ranged from 34.00 to 34.72 °C. In contrast, LT thermal exposures either need multiple temperature treatments^36,38^ or preliminary assessments to identify temperatures at which the strongest differential responses can be detected at the individual level. Moreover, it took over 197 h of chronic thermal stress to achieve the same bleaching response (ED25) detected within 5 h under acute thermal stress with the CBASS. In a restoration setting, there may be value in replacing chronic exposures with CBASS to improve scalability, especially if fine-scale physiological differences are conferred between the two approaches.

Across all phenotypic traits measured—photochemical efficiency of PSII (*F*_v_/*F*_m_), photosynthetic pigment (total chlorophyll [a + c_2_]), and host soluble protein—responses were most similar between the CBASS 34.3 °C treatment and the LT ocean warming (LT-OW; 30.5 °C) treatment. Importantly, the CBASS 34.3 °C treatment was closest to the nursery population’s upper bleaching threshold (ED50) and resolved most bleaching responses found under LT-OW. Yet, when each trait was independently compared among the two experimental treatments, bleaching responses did indeed differ, likely a result of different heating rates and heat accumulation under chronic and acute exposures. These findings contrast with other comparative thermal stress studies^36,38^, which observed comparable bleaching responses between treatments under ‘classic’ and acute thermal stress. It is notable that these ‘classic’ bleaching experiments refer to 10-14 d of treatment whereas the chronic heat treatment herein occurred over two months. Although two months of thermal stress would be considered more ecologically relevant than a 10-14 d exposure to achieve realistic measures of bleaching responses in longer-to-respond physiological traits, such as host protein concentrations^33,49^, it could be too long a time frame to achieve comparable results to the 18 h exposure of a CBASS experiment. Moreover, longer exposure periods can introduce photoacclimation to tank effects^35^ and confounds interpretations for heated corals, evident in the significant decline of the LT-Control *F*_v_/*F*_m_ values observed over the course of the experiment, despite ~ three weeks of post-collection acclimation. Since chlorophyll measures were not taken prior to the LT experiment, we cannot know whether chlorophyll values also acclimate to the experimental light regime, but it appears chlorophyll photophysiology adjusted differently than photochemistry given the significant effect of treatment for chlorophyll under the LT experiment. In contrast, there was not enough time for photoacclimation under acute exposures, as demonstrated by CBASS, where all treatments equal to or greater than the bleaching temperature of 34.3 °C were different from control values. Consequently, the varying heating durations between the two experiments, which represent the extreme ends of typical thermal stress exposures, likely exert different influences on the molecular, cellular, and physiological responses to thermal stress.

Most research utilizing CBASS to rapidly determine bleaching thresholds has focused on detecting population or regional-level distinctions^36,38^, with only one study incorporating colony-level assessment^15^. Hence, this study marks the first attempt to investigate thermal tolerance rankings among genotypes. Restoration programs with objectives to increase climate resilience would benefit from rapidly generated information regarding which nursery genotypes are likely to survive thermal stress. Instead of having clearly defined ‘winner’ genotype(s), genotype rankings were not straightforward and depended on the trait investigated and experiment type. Similar to the findings from Cunning et al.^15^, in each nursery population along FCR, there appears to be a mixture of tolerant and susceptible genotypes. The relative similarity in ED50 thresholds and bleaching response values across the genotypes examined herein support broad genetic and phenotypic variation sufficient for a diversity of responses against natural threats, especially if there is a lack of tradeoffs in disease resistance, fecundity, and/or growth^17,33^. Individuals of *A. cervicornis* in restoration nurseries are the resilient survivors from multiple decades of environmental stress in FCR so it is feasible that these common-gardened genotypes have similar bleaching outcomes. However, we did detect a consistent ‘loser’ genotype, UK12, between both studies. If 90% of restoration genotypes have similar thermal tolerances, as observed in this study, identifying those most susceptible may be a goal for restoration groups to potentially outplant these thermally sensitive genets to sites buffered from increasing water temperatures. Additionally, we recognize that ten genotypes is not fully representative of the nursery population and would need to repeat this experiment with more individuals to infer more fine-scale intrapopulation variation, as assessed in Cunning et al.^15^.

Although the ten *A. cervicornis* genotypes examined here have been common-gardened within an in situ nursery for a minimum of five years, fixed regional differences in coral source locations had an impact on bleaching responses, where ED50 values were higher for Upper Keys corals in comparison to Lower Keys genotypes. Most Upper Keys corals were collected from shallow patch reefs prior to entering MML’s restoration propagation pipeline in the Lower Keys (Table S1). Alternatively, Lower Keys genotypes were sourced from deeper midchannel reef sites and experience more stable temperatures annually, contributing to lower thermal thresholds in comparison to corals from shallow patch reef sites. Most thermal tolerance research attributes elevated thermotolerance in populations from shallower reef habitats that experience high thermal variability^3,18,50^, and the results from this study corroborate long-standing genetic effects on thermal tolerance. In contrast, Cunning et al.^15^ did not find any correlations between original source colony environmental parameters with nursery ED50 values, despite the six nurseries covering 300 km, but this could be attributed to satellite-derived SST information instead of capturing microhabitat variation at the in situ level^51^.

Patterns of fixed genetic effects of the source population also varied by experiment type and trait measured^36^. The LT-OW resolved coral source regional differences across all traits whereas CBASS indicated population-level differences for the relative change in *F*_v_/*F*_m_ only and in the opposite pattern as LT-OW. For example, Lower Keys sourced corals had lower *F*_v_/*F*_m_ values than Upper Keys corals under CBASS, but greater amounts of chlorophyll than Upper Keys corals under LT. Lower Keys corals are likely adapted to gradual changes in environmental stress and their ED50 suggests this population was more affected during acute heat stress but withstood the gradual chronic heat stress during LT exposures, resulting in chlorophyll values that were greater than Upper Keys corals. Within the Lower Keys population, genotype LK50 had one of the lowest *F*_v_/*F*_m_ under CBASS, but one of the highest amounts of chlorophyll under LT heat. Alternatively, Upper Keys-sourced corals could be considered adapted to the daily variability in their shallow environment^15^ and therefore would be less affected by the rapid heating rate under CBASS treatments, resulting in higher ED50 values than Lower Keys ED50s. For example, genotype UK12, which had the highest ED50 thermal threshold of 34.72 °C, had lower chlorophyll and host protein values in comparison to Lower Keys corals. Although sourced from shallow, variable reef habitats, it is possible that latitudinal differences in summer mean maxima could influence performance under chronic thermal stress and impair long-term survival after outplanting at restoration sites (but see^52^). Karp^53^ also found latitudinal source effects on thermal tolerance in Miami-Dade genotypes of *A. cervicornis* under a moderate thermal stress experiment (three weeks at 32.5 °C), although there was no relationship with source depth^15^.

Another possibility influencing the switch in genotype/population performance would be the observed discrepancy among the two fluorometric traits measured in the CBASS experiment, which could be attributed to the biological timing of measurable bleaching responses. Genotype UK12, which had the highest ED50 thermal threshold of 34.72 °C, was the lowest-ranked performer with regard to delayed bleaching traits (chlorophyll and protein). Some genotypes with higher *F*_v_/*F*_m_ retention (measured during CBASS) had lower chlorophyll values (measured at the end of CBASS) and vice versa (Fig. S3B). Recent work using *F*_v_/*F*_m_ values measured right after the CBASS thermal ramp profile before recovery has found thermal threshold differences in *F*_v_/*F*_m_ that corroborate experimental and natural bleaching responses in coral populations^18,36,38^. In contrast, we found a negative or no relationship between individual genotype ED50 values and the relative change in chlorophyll and protein among CBASS or LT exposures (Fig. 5). Voolstra et al.^36^ also found no correlations between experiment types for destructive bleaching responses (chlorophyll, protein) but did detect a correlation between *F*_v_/*F*_m_, thus arguing for that metric as a reliable indicator of bleaching responses. A line of evidence for the disparity in *F*_v_/*F*_m_ between this study and Voostra et al.^36^ could be attributed to population-level effects detected in Voolstra et al.^36^, where individuals from the protected reef site consistently performed better across all traits. Here, top-performing genotypes detected with *F*_v_/*F*_m_ during CBASS were not the top performers regarding bleaching outcomes, i.e., chlorophyll. This observed ‘switch’ in performance cautions conclusions drawn from utilizing only one photobiological trait to identify thermal tolerance^15^ and suggests that measurements should be conducted in parallel. Additionally, these results also suggest that CBASS may be useful for differentiating sub-populations among regions with different environmental conditions, but less so for differentiating genotype performance from corals within a common garden nursery.

The disparity among trait responses across experiment type and coral source region raises two important points, (1) is there a tradeoff between thermal tolerance and other physiological traits, and, (2) what is the best metric to assess heat stress tolerance? Initial comparative studies directly^38^ and indirectly^25^ demonstrated that CBASS can serve as a standardized approach to assess natural variation in thermal tolerance^37^. In this study, initial genotype or population resistance and physiological responses post-heat stress was mostly incongruent between acute and chronic thermal stress, despite having similar trait mean values when comparing bleaching treatments across experiment types. A complementary study by Nielsen et al.^48^ measured multiple physiological traits—*F*_v_/*F*_m_, color, chlorophyll, catalase, and protein—in *Acropora tenuis* from five reef sites and found that *F*_v_/*F*_m_, color, chlorophyll, and protein were all correlated and accounted for 43% of trait variation, similar to our findings in Fig. 2. Nielsen et al.^48^ also found a strong correlation between non-invasive metric(s) of *F*_v_/*F*_m_ and tissue color with laboratory-derived metrics, whereas we did not find a strong correlation between metrics within CBASS (Fig. S4) and between the experiment types (Fig. 5). This difference observed in this study is likely a result of the timing of measurements made, where Nielsen et al.^48^ sampled *F*_v_/*F*_m_ at the same time as all laboratory-derived metrics whereas we sampled *F*_v_/*F*_m_ at 0 h of recovery and laboratory-derived metrics at 12 h recovery similar to Voolstra et al.^36^. Thus, sampling traits at different time points provides contrasting snapshots of the underlying bleaching response in an individual coral, which could erroneously conclude that tradeoffs exist.

In Nielsen et al.^48^, each bleaching trait varied in its rate of decline under CBASS, signifying that integrative responses are occurring within or between different holobiont partners at different rates and thus challenge final bleaching response interpretations. Therefore, the particular trait measured, in addition to the timing of sampling, can confound CBASS^48^ and LT interpretations^25,38^. This study corroborates that the amount of decline in phenotypic traits can vary within^48^ and between^25,38^ heat stress exposure types. For example, *F*_v_/*F*_m_ was not related to total chlorophyll under CBASS in this study and *F*_v_/*F*_m_ was relatively stable throughout the Nielsen et al. study, up until 24 h post heat ramp, suggesting that *F*_v_/*F*_m_ as a bleaching response may not be as informative immediately after acute heat application in comparison to other higher-order traits such as tissue colour^48^. In contrast, other studies using stress tolerant corals such as *Stylophora pistillata* from protected sites in the Red Sea^36^ and *Siderastrea sidera* from nearshore reefs in Belize^54^ found that changes in *F*_v_/*F*_m_ values correspond strongly to other bleaching traits. Our study utilized a relatively thermally sensitive coral species maintained in a sandy bottom nursery. Despite different ocean basins and inherent thermal tolerance differences, this suggests that some bleaching trait metrics may not be as informative under a CBASS setting and may require chronic levels of stress for final bleaching outcomes. For example, host soluble protein concentrations are typically slower to respond, attributed to precursory molecular and physiological processes prior to measurable changes^49^ and do not display similar responses to acute heat stress like *F*_v_/*F*_m_ and chlorophyll (Fig. S5). Combining CBASS and LT studies with fine-scale temporal sampling^48^ and molecular analyses could help explain differences in physiological responses under different thermal exposures. Together, there are definite nuances in measuring and interpreting bleaching responses under CBASS and continued exploration of the methodology is needed to validate and resolve acute heat stress responses.

The increased frequency and severity of global thermal stress is threatening coral survival and ecosystem functioning, spurring the urgency to quantify coral thermal tolerance and identify tolerant individuals, populations, and species for conservation and restoration efforts rapidly and accurately. With an increased interest in high-throughput heat stress assays like the CBASS, it is prudent to conduct comparative studies between different length experimental thermal exposures to effectively corroborate natural bleaching responses and quantitatively compare the upper thermal limits of coral species, populations, and individuals^25,38^. Acute heat stress assays quickly screen multiple individuals to determine relative thermal tolerances and have resolved tolerance differences in many coral populations^4,15,18,23,26,30,36,38,40,55^. We assessed the applicability of using the acute CBASS to determine whether bleaching responses are similar in restoration nursery genotypes when compared to long-term thermal exposures. Our findings conclude that although CBASS can achieve bleaching responses more rapidly (time scalability), there were differences observed in bleaching traits between CBASS and LT experiments, some of which can be attributed to common-garden and/or fixed effects on nursery *A. cervicornis* and the timing of measurements. Among similarly ranked genotypes we did detect one poor performer (UK12), yet initial rankings from *F*_v_/*F*_m_ and ED50 threshold values measured right after acute heat were not comparable to endpoint bleaching response rankings (Fig. S6). Despite being common-gardened for > 5 yrs, our results suggest nursery corals sourced from the Lower Keys may be more adapted to chronic heat than Upper Keys corals while Upper Keys corals appeared to be initially less affected by acute heat. To better assess variation in heat tolerance and heat tolerant traits among restoration corals, we recommend comparing nursery with wild/outplanted genotypes as well as incorporating additional *F*_v_/*F*_m_ measurement timepoints that match other sampling measures for cross-comparison of bleaching phenotypes and detection of meaningful bleaching traits. Moreover, coupling physiological and molecular sampling would further our understanding of the timing of biological responses to make the most meaningful conclusions^56^, while correlating CBASS results to natural bleaching responses in restored populations can corroborate the utility of acute stress assays in a restoration context. Importantly, incorporating rapid, scalable non-invasive bleaching metrics^37,48,57^ to predict bleaching susceptibility alongside restoration interventions could improve restoration outcomes in lieu of declining coral cover in a warming world.

## Methods

### Coral collections

The long-term (LT) and acute (CBASS) experiments occurred at Mote Marine Laboratory’s International Center of Coral Reef Research and Restoration (MML-IC2R3; 24.6617° N, -81.4554° W) in Summerland Key, FL. On February 22^nd^, 2021, ten 5–7 cm replicate fragments from 10 unique genets of *Acropora cervicornis* were collected at random from coral nursery trees at ~ 4–5 m depth within MML’s Looe Key field nursery (24.56257° N, -81.40009° W). Unique genotypes were previously confirmed from SNPchip and 2bRAD analyses (https://galaxyproject.org/use/coral-snp/^58^; Table S1). Each genet was transported separately back to MML-IC2R3 where each replicate non-bifurcating fragment, or ramet, was glued to labeled ceramic reef plugs and held within an outdoor flow-through raceway (2.54 × 1.02 × 0.30 m) at MML’s Climate and Acidification Ocean Simulator (CAOS) facility. Coral ramets were maintained at ambient light (480 ± 100 µmol photon m^2^), temperature (27.5 °C), and pH_NBS_ (~ 8.1) levels for a three-week acclimatization period. Corals were broadcast fed biweekly using Golden Pearls (BulkReefSupply).

On June 11, 2021, 25 fragments (~ 5–6 cm long) of the same 10 genotypes of *A. cervicornis* (n = 250) were collected from MML’s in-situ Looe Key Nursery, glued to labeled ceramic plugs, and held in a temperature regulated deep raceway (2.54 × 1.93 × 0.61 m) within the CAOS system. The remaining corals (n = 240) were held with 4 submersible pumps (450 GPH) and flowing seawater from spigots at a rate of 240 L hr^-1^ for 5–7 days.

### Long-term and acute experimental exposures

For the LT exposure, ten 18.9 L flow-through glass aquaria (40.64 × 20.32 × 25.4 cm) were set up across two shallow treatment raceways in the CAOS system for the long-term exposure study^33,34^. Tanks contained a 120 GPH submersible pump (Dwyer) and received seawater from individual spigots at a rate of ~ 21 L hr^-1^ (Table S2). After the acclimation period, one coral ramet per genet was randomly assigned to a tank within the treatment raceways: heat (30.84 ± 0.61 °C) or control (26.92 ± 0.34 °C). In total, there were five replicate ramets of each coral genotype per treatment (ten corals per tank). Treatment conditions started on March 23, 2021, and were achieved incrementally by increasing temperature in the heat raceway by 0.5 °C per day for six days to reach 30.5 °C. After one month at 30.5 °C, temperatures were raised another 0.5 °C per day for two days to reach 31.5 °C and then held for two weeks until there was observable 50% bleaching in the heat treatment tanks, monitored using the CoralCard Health Chart and photochemical efficiency (*F*_v_/*F*_m_). The control temperature of 27.5 °C was maintained throughout. Temperature, salinity, dissolved oxygen, and pH_NBS_ were monitored with a YSI multi-parameter handheld (YSI Professional Series) daily at 0900 (GMT -5), light levels (PAR) were measured every 3–4 days using a Licor Handheld (Licor Li-1500 and Li-192 underwater quantum sensor; Table S2), and total alkalinity and dissolved inorganic carbon (*p*CO_2_, HCO_3-_, CO^2–^_3_, pH_T_ and aragonite saturation state were calculated using CO2Sys^59–61^ (Table S3) were measured to characterize water quality and carbonate parameters throughout the experiment.

From June 19 to June 21, 2021, three 18 h acute heat stress experiments were conducted using two Coral Bleaching Automated Stress Systems (CBASS^37^). Each CBASS system consisted of four 10 L flow-through tanks for a total of eight independently controlled treatments. Each tank was temperature regulated by two chillers (IceProbe Thermoelectric chillers, NovaTec) and a 150–200 W heater connected to a custom-built controller (Arduino Mega 2560) programmed to run independent, different thermal profiles for each tank. One replicate ramet from each genotype was randomly assigned to each tank and treatments were replicated over three days (n = 80 corals per day). The eight target temperatures treatments ranged from 27.5 to 37 °C, at 1.35 °C temperature increments, and were chosen based on CBASS experiments conducted on various nursery genotypes of *A. cervicornis*, where ~ 35–36 °C was identified to be the effective dose 50 (ED50^15^). All tanks were set to 27.5 °C at noon while corals were placed in tanks. At 1300 (GMT -4), temperatures ramped up to the respective target temperature over three hours (1300–1600), followed by a three-hour hold at max temperature (1600- 1900), a one-hour ramp down in temperature to 27.5 °C (1900–2000), and an overnight 11-h recovery at 27.5 °C (Fig. 1). Temperature treatments were randomized across tanks every day. Tanks were independently supplied with seawater from spigots at a rate of ~ 2 L hr^-1^ (~ 5 h renewal rate) until the end-of-hold, where inflow rates were increased to ~ 4 L hr^--1^ (~ 2.5 h renewal rate), and tanks were equipped with powerhead pumps to ensure sufficient water flow. LED aquarium lights (GalaxyHydro, Roleandro) provided ~ 300 mmol quanta m^-2^ s^-1^ on a 12 h:12 h light:dark cycle.

### Coral-algal phenotypes measured

Physiological measurements of photochemical efficiency of dark-adapted algal symbionts were taken at the beginning (March 15–22), middle (April 28–May 3), and end of two months of exposure (May 12–18, 2021). The maximum quantum yield of PSII, or *F*_v_/*F*_m_, was measured for each coral ramet using an Imaging Pulse Amplitude Modulation (I-PAM) fluorometer (Walz, Germany; settings: measuring light [ML] = 3, gain = 1–3, damping = 1, ML light intensity = 10, and ML width = 14). For the CBASS experiment, dark-adapted photochemical efficiency was measured following the end of the three-hour hold (2200 GMT-4). At the completion of both experiments, corals were individually placed in 7 oz Whirl Pak bags (NASCO), snap-frozen in liquid nitrogen, and then stored at -80 °C. Corals were processed for host soluble protein and total chlorophyll (chl *a* + c_2_) concentrations. All coral tissue was airbrushed from the skeleton with filtered seawater, tissue slurry volumes were recorded for scaling concentrations, and subsamples of tissue slurry were aliquoted for protein and chlorophyll extraction. Although three rounds of CBASS were conducted, the first replicate round experienced spurious coral mortality in all treatments during overnight recovery, and thus, samples could not be used for laboratory measurements of total chlorophyll and protein. Soluble protein was extracted in triplicate with Bradford reagent (VWR, Atlanta, GA) and measured on a microplate reader (Syngery H1, Agilent Technologies, Santa Clara, CA) at wavelength 595 nm using Bovine-Serum-Albumin for standard curves. Chlorophyll extraction was conducted in the dark using 90% acetone and 1 mm glass beads for algal cell shearing on a horizontal vortex adapter at maximum speed for 2 min. After 24 h, extracted chlorophyll was measured on a microplate reader at wavelengths 630 nm (chl c_2_), 664 nm (chl *a*), and 750 nm (turbidity), and absorbance values were applied to equations provided by Ritchie^62^ to calculate total chlorophyll (chl *a* + chl c_2_). Protein and chlorophyll concentrations were normalized to coral surface area, which was quantified using 3D photogrammetry and dense-point cloud mesh construction (AGI Metashape) and trimming in MeshLab^63^.

### Statistical analyses

To quantify the upper thermal thresholds for both the nursery population and each genotype, *F*_v_/*F*_m_ values measured at the beginning of CBASS recovery were used to derive ED50^38^. *F*_v_/*F*_m_ values were modeled to a dose–response curve using the R package *drc*^64,65^ to determine the x-value (temperature) at the inflection point of the curve where *F*_v_/*F*_m_ values are 50% lower than starting values, termed the ED50. Dose–response curves were fitted as three-parameter log-logistic functions to extract the ED50. A Welch’s one-way ANOVA was conducted on ED50 values to test for the fixed effect of source Region. Unlike CBASS, there was not a 50% decline in *F*_v_/*F*_m_ among all genotypes in the LT experiment, and therefore, ED50 values could not be determined, so ED25 was quantified instead^46^. *F*_v_/*F*_m_ measured at the beginning, middle, and end of the two-month exposure was modeled to a dose–response curve using the stress metric Degree Heating Hours (DHH^46,66^). DHH refers to the accumulation of heatloading hours over the regional bleaching threshold of 30.5 °C^11^. Log-logistic dose–response curves were fit separately to the LT and CBASS experiments, using all treatments from CBASS where the amount of heat accumulation per treatment served as the DHH value. From these curves, the ED25 parameter was calculated, representing the x-value where *F*_v_/*F*_m_ values were 25% lower than starting values^46^, and compared between the two experimental exposures.

The fixed effect of treatment and genotype for both LT and CBASS on all traits measured at the end of each experiment—*F*_v_/*F*_m_ (during for CBASS), total chlorophyll, and host protein—were compared using a PERMANOVA with the *vegan* package^67^. Data was standardized prior to conducting 999 permutations on Euclidean distance matrix to generate *p* values. Post-hoc analyses on the significant effect of temperature were used with the *pairwiseAdonis*^68^ with Bonferonni adjusted *p*-values. Pairwise comparisons among the significant interaction of experiment treatment and genotype could not be computed from the *pairwiseAdonis* package code, so the main effects were only examined.

Individual response variables of *F*_v_/*F*_m_, total chlorophyll, and host protein were analyzed in four ways. First, the fixed effect of treatment (levels = 9) was compared between experiments using Welch’s test, after confirming unequal variances. Next, linear mixed-effects models (LME) were used to compare the LT heat treatment with the CBASS 34.3 °C bleaching threshold treatment. Fixed effects of experiment (levels = 2) and genotype (levels = 10) and random effect of tank (levels = 21) were tested with ANOVA sum of squares type III in the *lmerTest* package^69^, and pairwise comparisons of significant effects were conducted using the *emmeans* function in the *emmeans* package^70^, adjusted for Tukey’s honestly significant difference (HSD). Third, LME models were again used to compare the fixed effects of treatment and genotype (accounting for tank as a random factor) within each experiment, and Tukey’s HSD post hoc pairwise comparisons were conducted for significant main effects using *emmeans*. For the LT experiment *F*_v_/*F*_m_ data, we also included a fixed effect of time to investigate photochemical changes from the start to the end of exposures. Lastly, the effect of coral source region (fixed) within each experiment was modeled with treatment (fixed) and tank (random) similarly to previously outlined analyses. The random effect of tank was analyzed using the *rand* function (*lmerTest* package) with single-term deletions. ANOVA assumptions of normality, homoscedasticity, and residual distribution were examined using the *sjPlot* function in the *sjPlot* package^71^. Total chlorophyll and host soluble protein were square-root transformed to improve model fit.

The change in each trait, calculated as heat—control, was correlated between the LT heat and CBASS 34.3 °C ED50 threshold treatment. Since photoacclimation was detected in the LT Control *F*_v_/*F*_m_ data, *F*_v_/*F*_m_ loss was calculated from heat final and initial measurements, instead of between treatments. Linear relationships and Pearson correlation coefficients of trait means by genotype were determined for the regression LT heat ~ CBASS 34.3 °C. Additionally, we examined the relationship between bleaching traits (*F*_v_/*F*_m_ and total chlorophyll*)* which were measured at different times during both experiments. Total chlorophyll was regressed against *F*_v_/*F*_m_ to determine linear relationships and correlation coefficients within LT heat, CBASS 34.3 °C, and CBASS 35.6 °C. Within CBASS 34.3 °C, we also correlated each physiological trait to one another using the *cor* function (corrplot package^72^).

### Supplementary Information

Supplementary Information.
