# Supplementary material for: Assessing acute thermal assays as a rapid screening tool for coral restoration
Source: Sci Rep. 2024 Jan 22;14:1898. doi: 10.1038/s41598-024-51944-5 (PMC10803358; doi:10.1038/s41598-024-51944-5)
Supplement: Supplementary file 1 — Supplementary Information. [file 41598_2024_51944_MOESM1_ESM.pdf]

RESULTS

Within-experiment bleaching responses

Fv/Fm values taken during CBASS became significantly lower with each subsequent treatment over 32.9 °C (T $p < 0.0001$; Fig. S2A), and treatments 32.9 °C and lower were not different from one another. *Fv/Fm* declined by 49% under 34.3 °C and 82% under 35.6 °C compared to the control treatment of 27.5 °C. Under the LT experiment, LT-OW end *Fv/Fm* values decreased by 42% compared to initial *Fv/Fm* values. LT-OW *Fv/Fm* initial values were on average 1.7-fold higher than end values (Welch's $p < 0.0001$). LT-Control end *Fv/Fm* values were significantly higher than LT-OW end values, but only for Upper Keys genotypes (T x G $p < 0.05$). CBASS total chlorophyll (µg/cm^2; square-root transformed) in the 34.3 °C treatment declined by 45% and 85% declines were detected in the 35.6 °C treatment in comparison to the CBASS control (T $p = 0.001$; Fig. S2B). Under the LT experiment, there was a significant effect of treatment on all genotypes' total chlorophyll (TxG $p < 0.0001$; Fig. S2B), where chlorophyll declined by 92% under LT-OW (0.94 ± 0.44 µg/cm^2) compared to LT-Control (10.93 ± 3.46 µg/cm^2). Host protein (µg/cm^2; square-root transformed) was much more variable and did not differ across CBASS treatments (T $p > 0.05$; Fig. S2C). Protein values ranged from 22.82 ± 11.29 µg/cm^2 (35.6 °C) to 50.23 ± 11.9 µg/cm^2 (27.5 °C) in the CBASS experiment. In the LT experiment, host protein declined by 58% in the LT-OW compared to LT-Control (T $p < 0.001$; Fig. S2C), although there was no treatment effect for genotypes LK31, LK50, and UK70 (TxG $p > 0.05$).

TABLES & FIGURES

Table S1. Coral Genotype Source Location

Genotype	Habitat	Site Name	Latitude	Longitude
LK31	Offshore	Porkys	24.548	-81.457
LK41	Offshore	Haslun's	24.553	-81.438
LK50	Midchannel	1.8nm WSW of Wonderland	24.549	-81.533
LK62	Midchannel	Super Stag	24.530	-81.641
LK7	Midchannel	Wonderland	24.560	-81.501
UK12	Patch Reef	Plantation Key	24.966	-80.489
UK19	Patch Reef	Tavernier	24.988	-80.478
UK70	Patch Reef	Inshore from Molasses Trench	25.013	-80.407
UK76	Forereef	Lower Matecumbe	24.822	-80.656
UK80	Patch Reef	Shallow patch reef near U10	25.021	-80.401

Table S2. Summary of daily mean ± SD water quality parameters for the long-term (LT) Control and Ocean Warming (OW) treatment tanks (Temperature, DO [Dissolved Oxygen], Salinity, pH, Photosynthetically Active Radiation [PAR], Flow).

Treatment	Temp (°C)	DO (mg L^{-1})	DO (%)	Salinity (ppt)	pH_{NBS}	PAR (µmol m^{-2} sec^{-1})	Flow (L hr^{-1})
Control	26.92 ± 0.34	5.67 ± 0.28	87.58 ± 4.25	37.40 ± 0.53	8.08 ± 0.05	423.98 ± 216.24	21.92 ± 0.63
OW	30.84 ± 0.61	5.54 ± 0.34	91.02 ± 5.47	37.38 ± 0.63	8.09 ± 0.05	418.51 ± 218.97	21.86 ± 0.64

Table S3. Summary of carbonate chemistry parameters for the long-term experiment. Water was collected from the main holding tank ("Header Tank") and poisoned with mercuric chloride twice a week before analysis (pH_T [total scale], pCO2 [partial pressure of CO_2], DIC [Dissolved Inorganic Carbon], HCO_{3-} [Bicarbonate], CO^{2-}_3 [Carbonate], Ωar [Saturation state of seawater with respect to aragonite], TA [Total Alkalinity]).

	pH_T	pCO_2	DIC	HCO_{3-}	CO^{2-}_3	Ωar	TA
Header Tank	7.99 ± 0.08	490.29 ± 111.89	2084.04 ± 69.98	1848.71 ± 79.20	222.32 ± 35.79	3.51 ± 0.57	2397.92 ± 80.95

Table S4. Post-hoc Tukey HSD comparisons between genotype of *Fv/Fm* values under heat (LT-OW [31.5°C] and CBASS 34.3°C) treatments.

CBASS 34.3°C						LT-OW					
**Contrast**	**estimate**	**SE**	**df**	**t.ratio**	**p.value**	**Contrast**	**estimate**	**SE**	**df**	**t.ratio**	**p.value**
LK31 - LK41	-0.114	0.035	52	-3.260	0.056	**LK31 - LK41**	**0.110**	**0.027**	**52**	**4.035**	**0.0064**
LK31 - LK50	-0.026	0.035	52	-0.751	0.999	LK31 - LK50	0.009	0.027	52	0.334	1
LK31 - LK62	-0.039	0.035	52	-1.122	0.9801	LK31 - LK62	0.001	0.027	52	0.028	1
**LK31 - LK7**	**-0.137**	**0.035**	**52**	**-3.916**	**0.0091**	LK31 - LK7	0.022	0.027	52	0.814	0.9981
**LK31 - UK12**	**-0.148**	**0.035**	**52**	**-4.220**	**0.0036**	**LK31 - UK12**	**0.167**	**0.027**	**52**	**6.139**	**<.0001**
**LK31 - UK19**	**-0.143**	**0.035**	**52**	**-4.078**	**0.0056**	LK31 - UK19	0.039	0.027	52	1.421	0.9149
LK31 - UK70	-0.111	0.035	52	-3.156	0.0726	LK31 - UK70	0.078	0.027	52	2.887	0.1354
**LK31 - UK76**	**-0.131**	**0.040**	**52.4**	**-3.316**	**0.0484**	**LK31 - UK76**	**0.098**	**0.029**	**52.2**	**3.378**	**0.0412**
LK31 - UK80	-0.051	0.035	52	-1.445	0.9068	LK31 - UK80	0.080	0.027	52	2.956	0.1162
LK41 - LK50	0.088	0.035	52	2.509	0.2877	**LK41 - LK50**	**-0.101**	**0.027**	**52**	**-3.701**	**0.017**
LK41 - LK62	0.075	0.035	52	2.139	0.51	**LK41 - LK62**	**-0.109**	**0.027**	**52**	**-4.007**	**0.0069**
LK41 - LK7	-0.023	0.035	52	-0.656	0.9996	LK41 - LK7	-0.088	0.027	52	-3.221	0.0618
LK41 - UK12	-0.034	0.035	52	-0.960	0.9933	LK41 - UK12	0.057	0.027	52	2.105	0.5328
LK41 - UK19	-0.029	0.035	52	-0.817	0.998	LK41 - UK19	-0.071	0.027	52	-2.614	0.2375
LK41 - UK70	0.004	0.035	52	0.105	1	LK41 - UK70	-0.031	0.027	52	-1.147	0.9769
LK41 - UK76	-0.017	0.040	52.4	-0.430	1	LK41 - UK76	-0.012	0.029	52.2	-0.404	1
LK41 - UK80	0.064	0.035	52	1.816	0.7226	LK41 - UK80	-0.029	0.027	52	-1.079	0.9848
LK50 - LK62	-0.013	0.035	52	-0.371	1	LK50 - LK62	-0.008	0.027	52	-0.306	1
LK50 - LK7	-0.111	0.035	52	-3.165	0.0709	LK50 - LK7	0.013	0.027	52	0.480	1
**LK50 - UK12**	**-0.122**	**0.035**	**52**	**-3.469**	**0.0324**	**LK50 - UK12**	**0.158**	**0.027**	**52**	**5.806**	**<.0001**
**LK50 - UK19**	**-0.117**	**0.035**	**52**	**-3.327**	**0.0472**	LK50 - UK19	0.030	0.027	52	1.087	0.9839
LK50 - UK70	-0.084	0.035	52	-2.405	0.3442	LK50 - UK70	0.069	0.027	52	2.554	0.2656
LK50 - UK76	-0.105	0.040	52.4	-2.651	0.2207	LK50 - UK76	0.089	0.029	52.2	3.066	0.0901
LK50 - UK80	-0.024	0.035	52	-0.694	0.9994	LK50 - UK80	0.071	0.027	52	2.622	0.2336
LK62 - LK7	-0.098	0.035	52	-2.795	0.1653	LK62 - LK7	0.021	0.027	52	0.785	0.9985
LK62 - UK12	-0.109	0.035	52	-3.099	0.0834	**LK62 - UK12**	**0.166**	**0.027**	**52**	**6.111**	**<.0001**
LK62 - UK19	-0.104	0.035	52	-2.956	0.1162	LK62 - UK19	0.038	0.027	52	1.393	0.9239
LK62 - UK70	-0.071	0.035	52	-2.034	0.58	LK62 - UK70	0.078	0.027	52	2.859	0.144
LK62 - UK76	-0.092	0.040	52.4	-2.323	0.392	**LK62 - UK76**	**0.097**	**0.029**	**52.2**	**3.352**	**0.0441**
LK62 - UK80	-0.011	0.035	52	-0.323	1	LK62 - UK80	0.080	0.027	52	2.928	0.1238
LK7 - UK12	-0.011	0.035	52	-0.304	1	**LK7 - UK12**	**0.145**	**0.027**	**52**	**5.326**	**0.0001**
LK7 - UK19	-0.006	0.035	52	-0.162	1	LK7 - UK19	0.017	0.027	52	0.608	0.9998
LK7 - UK70	0.027	0.035	52	0.760	0.9988	LK7 - UK70	0.056	0.027	52	2.074	0.5533
LK7 - UK76	0.006	0.040	52.4	0.150	1	LK7 - UK76	0.076	0.029	52.2	2.616	0.2364
LK7 - UK80	0.087	0.035	52	2.471	0.3076	LK7 - UK80	0.058	0.027	52	2.143	0.5075
UK12 - UK19	0.005	0.035	52	0.143	1	**UK12 - UK19**	**-0.128**	**0.027**	**52**	**-4.718**	**0.0007**
UK12 - UK70	0.037	0.035	52	1.065	0.9861	UK12 - UK70	-0.088	0.027	52	-3.252	0.0572
UK12 - UK76	0.017	0.040	52.4	0.420	1	UK12 - UK76	-0.069	0.029	52.2	-2.376	0.3605
UK12 - UK80	0.097	0.035	52	2.776	0.172	UK12 - UK80	-0.086	0.027	52	-3.183	0.0679
UK19 - UK70	0.032	0.035	52	0.922	0.995	UK19 - UK70	0.040	0.027	52	1.466	0.8991
UK19 - UK76	0.012	0.040	52.4	0.293	1	UK19 - UK76	0.059	0.029	52.2	2.046	0.5719
UK19 - UK80	0.092	0.035	52	2.633	0.2289	UK19 - UK80	0.042	0.027	52	1.535	0.8716
UK70 - UK76	-0.021	0.040	52.4	-0.523	0.9999	UK70 - UK76	0.019	0.029	52.2	0.672	0.9996
UK70 - UK80	0.060	0.035	52	1.711	0.7843	UK70 - UK80	0.002	0.027	52	0.069	1
UK76 - UK80	0.081	0.040	52.4	2.037	0.5779	UK76 - UK80	-0.018	0.029	52.2	-0.607	0.9998

Table S5. Post-hoc Tukey HSD comparisons between genotype of Total Chlorophyll values under heat (LT-OW [31.5°C] and CBASS 34.3°C) treatments.

CBASS 34.3°C						LT-OW					
**Contrast**	**estimate**	**SE**	**df**	**t.ratio**	**p.value**	**Contrast**	**estimate**	**SE**	**df**	**t.ratio**	**p.value**
LK31 - LK41	0.685	0.210	29	3.263	0.0711	LK31 - LK41	-0.026	0.178	29.7	-0.145	1
LK31 - LK50	0.415	0.210	29	1.978	0.62	LK31 - LK50	-0.471	0.157	29.4	-2.996	0.1243
**LK31 - LK62**	**0.827**	**0.210**	**29**	**3.940**	**0.0144**	LK31 - LK62	-0.327	0.163	29.4	-2.004	0.6032
LK31 - LK7	0.047	0.210	29	0.224	1	LK31 - LK7	-0.271	0.177	29.6	-1.525	0.8712
**LK31 - UK12**	**1.204**	**0.210**	**29**	**5.734**	**0.0001**	**LK31 - UK12**	**0.740**	**0.157**	**29.4**	**4.704**	**0.002**
LK31 - UK19	0.451	0.210	29	2.150	0.5103	LK31 - UK19	0.170	0.195	29.2	0.871	0.9963
LK31 - UK70	-0.226	0.210	29	-1.078	0.9833	LK31 - UK70	0.004	0.176	29.4	0.025	1
LK31 - UK76	0.437	0.210	29	2.083	0.5524	LK31 - UK76	0.088	0.177	29.6	0.495	1
LK31 - UK80	0.022	0.210	29	0.103	1	LK31 - UK80	0.142	0.171	29	0.826	0.9975
LK41 - LK50	-0.270	0.210	29	-1.285	0.9493	LK41 - LK50	-0.445	0.157	29.4	-2.835	0.1708
LK41 - LK62	0.142	0.210	29	0.677	0.9995	LK41 - LK62	-0.301	0.162	29.2	-1.853	0.698
LK41 - LK7	-0.638	0.210	29	-3.038	0.1146	LK41 - LK7	-0.245	0.179	29.8	-1.368	0.9275
LK41 - UK12	0.519	0.210	29	2.472	0.3237	**LK41 - UK12**	**0.766**	**0.157**	**29.4**	**4.875**	**0.0012**
LK41 - UK19	-0.234	0.210	29	-1.113	0.9793	LK41 - UK19	0.196	0.196	29.4	0.998	0.9901
**LK41 - UK70**	**-0.911**	**0.210**	**29**	**-4.340**	**0.0052**	LK41 - UK70	0.030	0.183	30.1	0.166	1
LK41 - UK76	-0.248	0.210	29	-1.179	0.9699	LK41 - UK76	0.114	0.179	29.8	0.636	0.9997
LK41 - UK80	-0.663	0.210	29	-3.160	0.0889	LK41 - UK80	0.168	0.178	29.7	0.942	0.9935
LK50 - LK62	0.412	0.210	29	1.962	0.6297	LK50 - LK62	0.144	0.143	29.2	1.011	0.9892
LK50 - LK7	-0.368	0.210	29	-1.753	0.7571	LK50 - LK7	0.201	0.157	29.4	1.275	0.9516
**LK50 - UK12**	**0.789**	**0.210**	**29**	**3.757**	**0.0226**	**LK50 - UK12**	**1.211**	**0.133**	**29**	**9.118**	**<.0001**
LK50 - UK19	0.036	0.210	29	0.172	1	**LK50 - UK19**	**0.641**	**0.182**	**29.5**	**3.526**	**0.0387**
LK50 - UK70	-0.641	0.210	29	-3.055	0.1106	LK50 - UK70	0.476	0.157	29.4	3.029	0.1162
LK50 - UK76	0.022	0.210	29	0.106	1	**LK50 - UK76**	**0.559**	**0.157**	**29.4**	**3.554**	**0.0363**
LK50 - UK80	-0.394	0.210	29	-1.875	0.6848	**LK50 - UK80**	**0.613**	**0.157**	**29.4**	**3.897**	**0.0158**
**LK62 - LK7**	**-0.780**	**0.210**	**29**	**-3.716**	**0.025**	LK62 - LK7	0.056	0.168	29.9	0.335	1
LK62 - UK12	0.377	0.210	29	1.794	0.7333	**LK62 - UK12**	**1.067**	**0.143**	**29.2**	**7.481**	**<.0001**
LK62 - UK19	-0.376	0.210	29	-1.790	0.7357	LK62 - UK19	0.497	0.187	29.4	2.653	0.2384
**LK62 - UK70**	**-1.053**	**0.210**	**29**	**-5.017**	**0.0009**	LK62 - UK70	0.331	0.168	29.8	1.976	0.6206
LK62 - UK76	-0.390	0.210	29	-1.857	0.6959	LK62 - UK76	0.415	0.168	29.9	2.466	0.3254
**LK62 - UK80**	**-0.806**	**0.210**	**29**	**-3.837**	**0.0186**	LK62 - UK80	0.469	0.163	29.4	2.872	0.1591
**LK7 - UK12**	**1.157**	**0.210**	**29**	**5.510**	**0.0002**	**LK7 - UK12**	**1.010**	**0.157**	**29.4**	**6.424**	**<.0001**
LK7 - UK19	0.404	0.210	29	1.925	0.6531	LK7 - UK19	0.440	0.195	29.3	2.253	0.446
LK7 - UK70	-0.273	0.210	29	-1.302	0.9452	LK7 - UK70	0.275	0.176	29.4	1.564	0.8544
LK7 - UK76	0.390	0.210	29	1.859	0.6944	LK7 - UK76	0.358	0.171	29	2.090	0.5479
LK7 - UK80	-0.025	0.210	29	-0.121	1	LK7 - UK80	0.412	0.177	29.6	2.324	0.4038
**UK12 - UK19**	**-0.753**	**0.210**	**29**	**-3.585**	**0.0341**	UK12 - UK19	-0.570	0.182	29.5	-3.137	0.0926
**UK12 - UK70**	**-1.430**	**0.210**	**29**	**-6.812**	**<.0001**	**UK12 - UK70**	**-0.735**	**0.157**	**29.4**	**-4.683**	**0.0021**
**UK12 - UK76**	**-0.767**	**0.210**	**29**	**-3.651**	**0.0292**	**UK12 - UK76**	**-0.652**	**0.157**	**29.4**	**-4.145**	**0.0084**
**UK12 - UK80**	**-1.182**	**0.210**	**29**	**-5.631**	**0.0002**	**UK12 - UK80**	**-0.598**	**0.157**	**29.4**	**-3.804**	**0.0199**
UK19 - UK70	-0.678	0.210	29	-3.227	0.0768	UK19 - UK70	-0.165	0.201	29.6	-0.823	0.9976
UK19 - UK76	-0.014	0.210	29	-0.066	1	UK19 - UK76	-0.082	0.195	29.3	-0.419	1
UK19 - UK80	-0.430	0.210	29	-2.047	0.5758	UK19 - UK80	-0.028	0.195	29.2	-0.144	1
UK70 - UK76	0.664	0.210	29	3.161	0.0886	UK70 - UK76	0.083	0.176	29.4	0.474	1
UK70 - UK80	0.248	0.210	29	1.181	0.9697	UK70 - UK80	0.137	0.176	29.4	0.777	0.9984
UK76 - UK80	-0.416	0.210	29	-1.980	0.6183	UK76 - UK80	0.054	0.177	29.6	0.303	1

Table S6. Post-hoc Tukey HSD comparisons between genotype Host Protein values under heat (LT-OW [31.5°C] and CBASS 34.3°C) treatments.

CBASS 34.3°C						LT-OW					
**Contrast**	**estimate**	**SE**	**df**	**t.ratio**	**p.value**	**Contrast**	**estimate**	**SE**	**df**	**t.ratio**	**p.value**
LK31 - LK41	-1.647	1.291	29.1	-1.276	0.9514	LK31 - LK41	1.077	1.197	29.7	0.899	0.9953
LK31 - LK50	0.889	1.291	29.1	0.689	0.9994	LK31 - LK50	-0.283	0.967	30.3	-0.293	1
LK31 - LK62	0.657	1.291	29.1	0.509	0.9999	LK31 - LK62	1.307	1.003	30.1	1.303	0.9452
LK31 - LK7	-0.290	1.291	29.1	-0.224	1	LK31 - LK7	0.362	1.088	30.5	0.333	1
LK31 - UK12	2.285	1.291	29.1	1.770	0.7477	**LK31 - UK12**	**3.822**	**0.967**	**30.3**	**3.953**	**0.0133**
LK31 - UK19	-0.122	1.291	29.1	-0.095	1	LK31 - UK19	1.521	1.197	29.7	1.270	0.9528
LK31 - UK70	-1.159	1.291	29.1	-0.898	0.9953	LK31 - UK70	-0.348	0.999	29.7	-0.349	1
LK31 - UK76	-0.375	1.291	29.1	-0.290	1	LK31 - UK76	0.996	1.088	30.5	0.916	0.9947
LK31 - UK80	1.709	1.291	29.1	1.324	0.9397	LK31 - UK80	1.448	1.054	29.1	1.374	0.9256
LK41 - LK50	2.536	1.291	29.1	1.965	0.6282	LK41 - LK50	-1.359	1.118	30.5	-1.215	0.964
LK41 - LK62	2.305	1.291	29.1	1.785	0.7386	LK41 - LK62	0.231	1.152	30.4	0.200	1
LK41 - LK7	1.358	1.291	29.1	1.052	0.9858	LK41 - LK7	-0.714	1.199	29.9	-0.596	0.9998
LK41 - UK12	3.932	1.291	29.1	3.046	0.1127	LK41 - UK12	2.745	1.118	30.5	2.454	0.331
LK41 - UK19	1.525	1.291	29.1	1.181	0.9696	LK41 - UK19	0.444	1.291	29.1	0.344	1
LK41 - UK70	0.489	1.291	29.1	0.378	1	LK41 - UK70	-1.425	1.147	30.1	-1.243	0.9587
LK41 - UK76	1.273	1.291	29.1	0.986	0.9909	LK41 - UK76	-0.080	1.199	29.9	-0.067	1
LK41 - UK80	3.356	1.291	29.1	2.600	0.262	LK41 - UK80	0.372	1.197	29.7	0.311	1
LK50 - LK62	-0.232	1.291	29.1	-0.179	1	LK50 - LK62	1.590	0.876	29.6	1.816	0.7204
LK50 - LK7	-1.179	1.291	29.1	-0.913	0.9947	LK50 - LK7	0.645	0.967	30.3	0.667	0.9995
LK50 - UK12	1.396	1.291	29.1	1.081	0.9829	**LK50 - UK12**	**4.105**	**0.816**	**29.1**	**5.027**	**0.0008**
LK50 - UK19	-1.011	1.291	29.1	-0.783	0.9983	LK50 - UK19	1.803	1.118	30.5	1.612	0.8321
LK50 - UK70	-2.048	1.291	29.1	-1.586	0.844	LK50 - UK70	-0.066	0.878	29.9	-0.075	1
LK50 - UK76	-1.264	1.291	29.1	-0.979	0.9913	LK50 - UK76	1.279	0.967	30.3	1.323	0.9402
LK50 - UK80	0.820	1.291	29.1	0.635	0.9997	LK50 - UK80	1.731	0.967	30.3	1.791	0.7357
LK62 - LK7	-0.947	1.291	29.1	-0.734	0.999	LK62 - LK7	-0.945	1.031	31.2	-0.917	0.9947
LK62 - UK12	1.627	1.291	29.1	1.260	0.9548	LK62 - UK12	2.514	0.876	29.6	2.872	0.1588
LK62 - UK19	-0.780	1.291	29.1	-0.604	0.9998	LK62 - UK19	0.213	1.152	30.4	0.185	1
LK62 - UK70	-1.816	1.291	29.1	-1.407	0.9153	LK62 - UK70	-1.656	0.940	30.6	-1.762	0.7525
LK62 - UK76	-1.032	1.291	29.1	-0.799	0.998	LK62 - UK76	-0.311	1.031	31.2	-0.302	1
LK62 - UK80	1.052	1.291	29.1	0.815	0.9977	LK62 - UK80	0.141	1.003	30.1	0.141	1
LK7 - UK12	2.574	1.291	29.1	1.994	0.6095	**LK7 - UK12**	**3.460**	**0.967**	**30.3**	**3.579**	**0.0335**
LK7 - UK19	0.168	1.291	29.1	0.130	1	LK7 - UK19	1.158	1.199	29.9	0.966	0.9922
LK7 - UK70	-0.869	1.291	29.1	-0.673	0.9995	LK7 - UK70	-0.711	0.998	29.6	-0.712	0.9992
LK7 - UK76	-0.085	1.291	29.1	-0.066	1	LK7 - UK76	0.634	1.054	29.1	0.602	0.9998
LK7 - UK80	1.999	1.291	29.1	1.548	0.8612	LK7 - UK80	1.086	1.088	30.5	0.999	0.9901
UK12 - UK19	-2.407	1.291	29.1	-1.864	0.6912	UK12 - UK19	-2.301	1.118	30.5	-2.057	0.5685
UK12 - UK70	-3.443	1.291	29.1	-2.667	0.2331	**UK12 - UK70**	**-4.170**	**0.878**	**29.9**	**-4.749**	**0.0017**
UK12 - UK76	-2.659	1.291	29.1	-2.060	0.5673	UK12 - UK76	-2.826	0.967	30.3	-2.923	0.1428
UK12 - UK80	-0.576	1.291	29.1	-0.446	1	UK12 - UK80	-2.373	0.967	30.3	-2.455	0.3311
UK19 - UK70	-1.037	1.291	29.1	-0.803	0.998	UK19 - UK70	-1.869	1.147	30.1	-1.630	0.8233
UK19 - UK76	-0.253	1.291	29.1	-0.196	1	UK19 - UK76	-0.524	1.199	29.9	-0.437	1
UK19 - UK80	1.831	1.291	29.1	1.418	0.9114	UK19 - UK80	-0.072	1.197	29.7	-0.060	1
UK70 - UK76	0.784	1.291	29.1	0.607	0.9998	UK70 - UK76	1.345	0.998	29.6	1.347	0.9335
UK70 - UK80	2.868	1.291	29.1	2.222	0.4655	UK70 - UK80	1.797	0.999	29.7	1.798	0.7312
UK76 - UK80	2.084	1.291	29.1	1.614	0.8308	UK76 - UK80	0.452	1.088	30.5	0.416	1

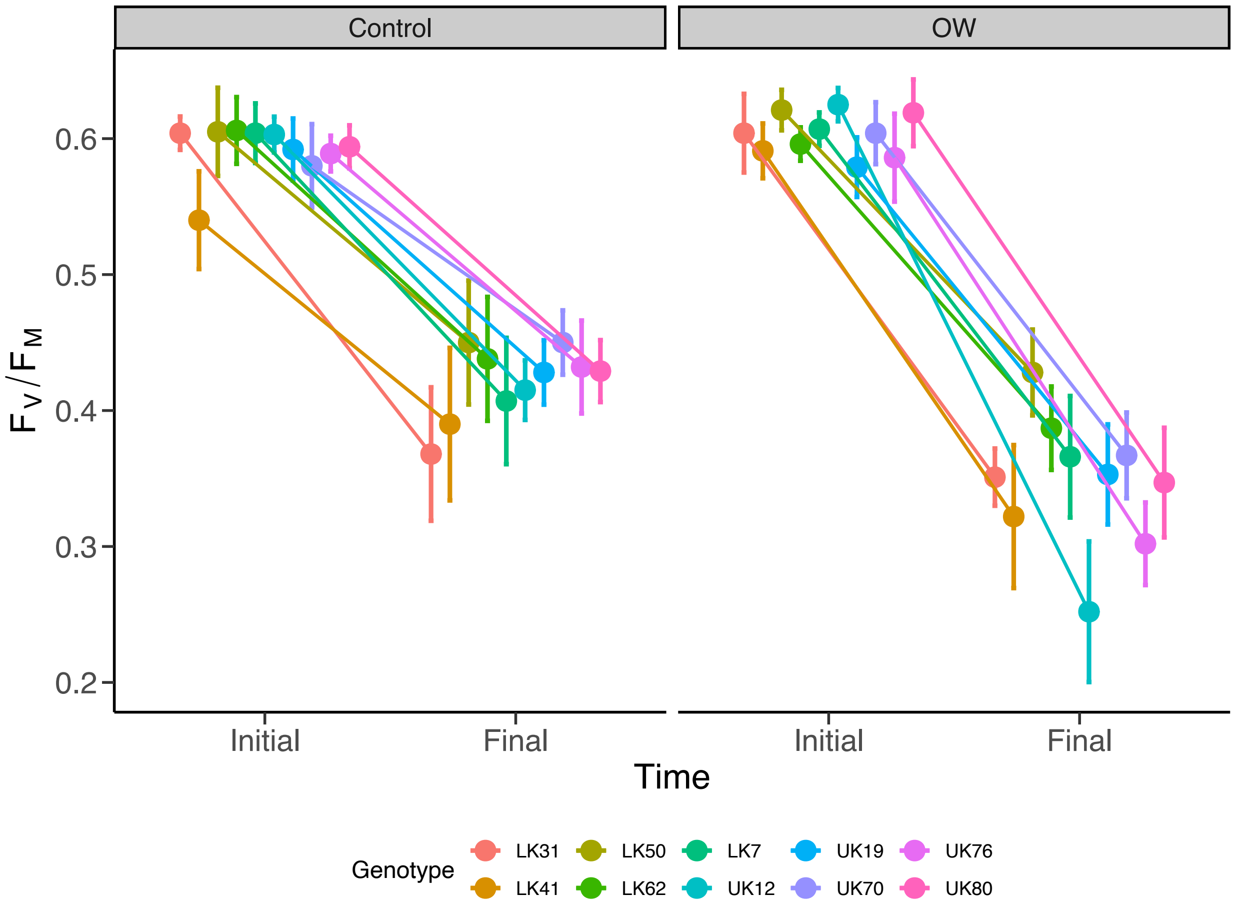

Figure S1. Evidence for photoacclimation over time under LT exposure. Mean (± 95% CI) values for each genotype are faceted by treatment (Control, OW).

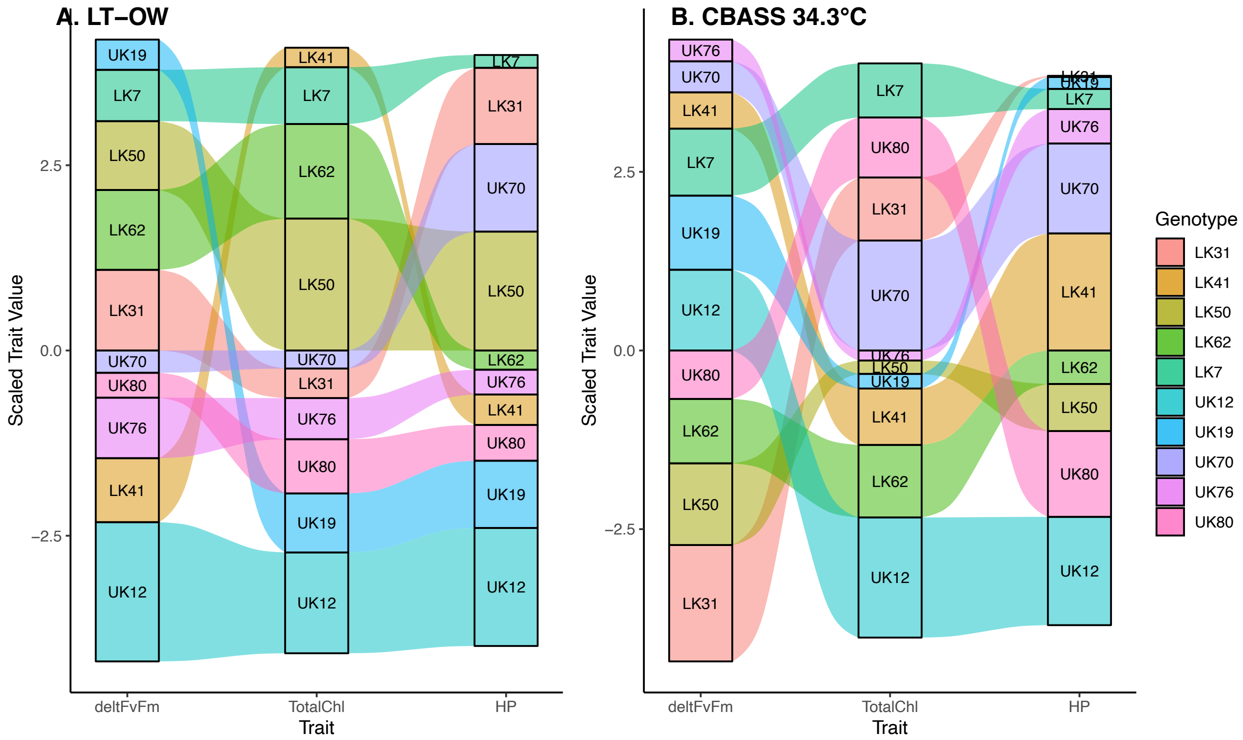

Figure S2. Alluvial plot for ranked genotypic scaled trait values across bleaching traits measured under (A) LT-OW and (B) CBASS 34.3 °C. Strata (boxes) are in descending order and sized based on scaled trait value between adjacent genotypes (colors).

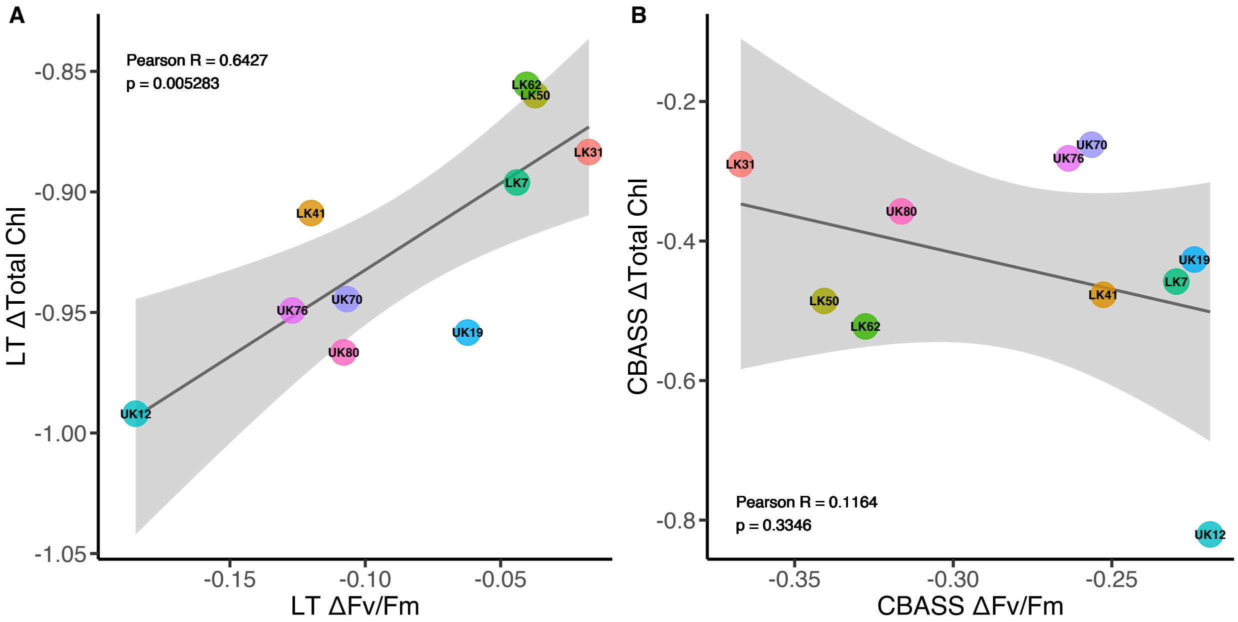

Figure S3. Correlations of the linear relationship between the relative change (Δ) in F_v/F_m and total chlorophyll (μg cm^{-2}) for (A) LT-OW and (B) CBASS 34.3 °C treatments. Linear regression results are reported and the gray-shaded area represents 95% confidence intervals.

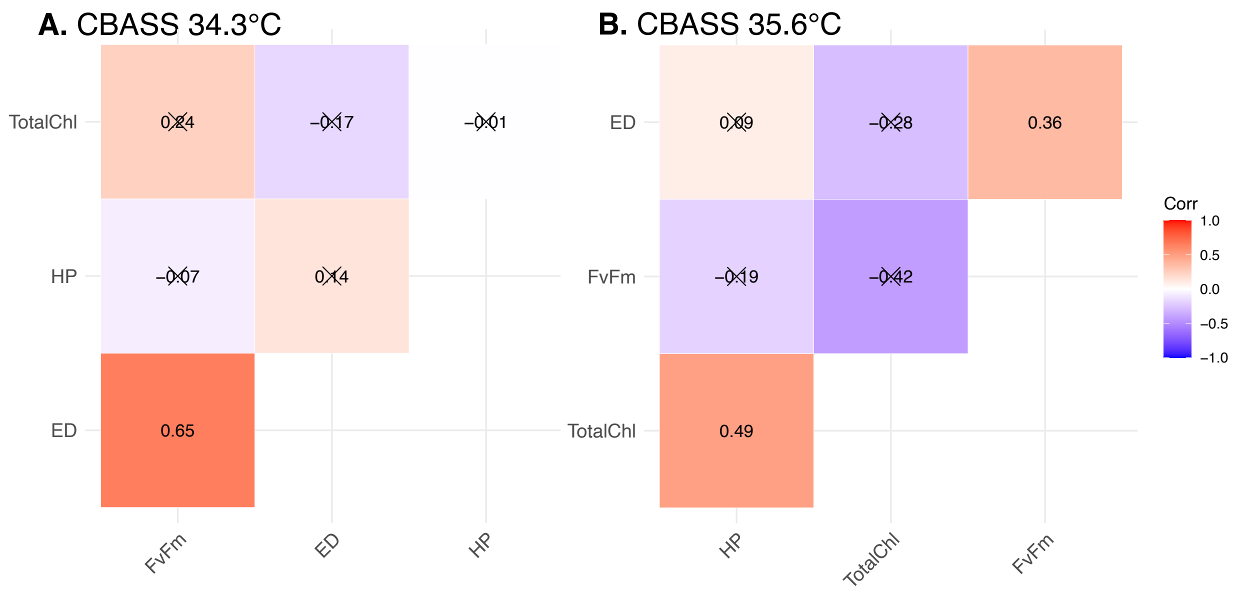

Figure S4. Correlation plot matrix among all bleaching traits (ED50 [ED], F_v/F_m, total chlorophyll [TotalChl], and host protein [HP]) for the CBASS treatments of (A) 34.3 °C and (B) 35.6 °C. Numbers inside boxes represent Pearson correlation coefficients and "X" represents non-significance.

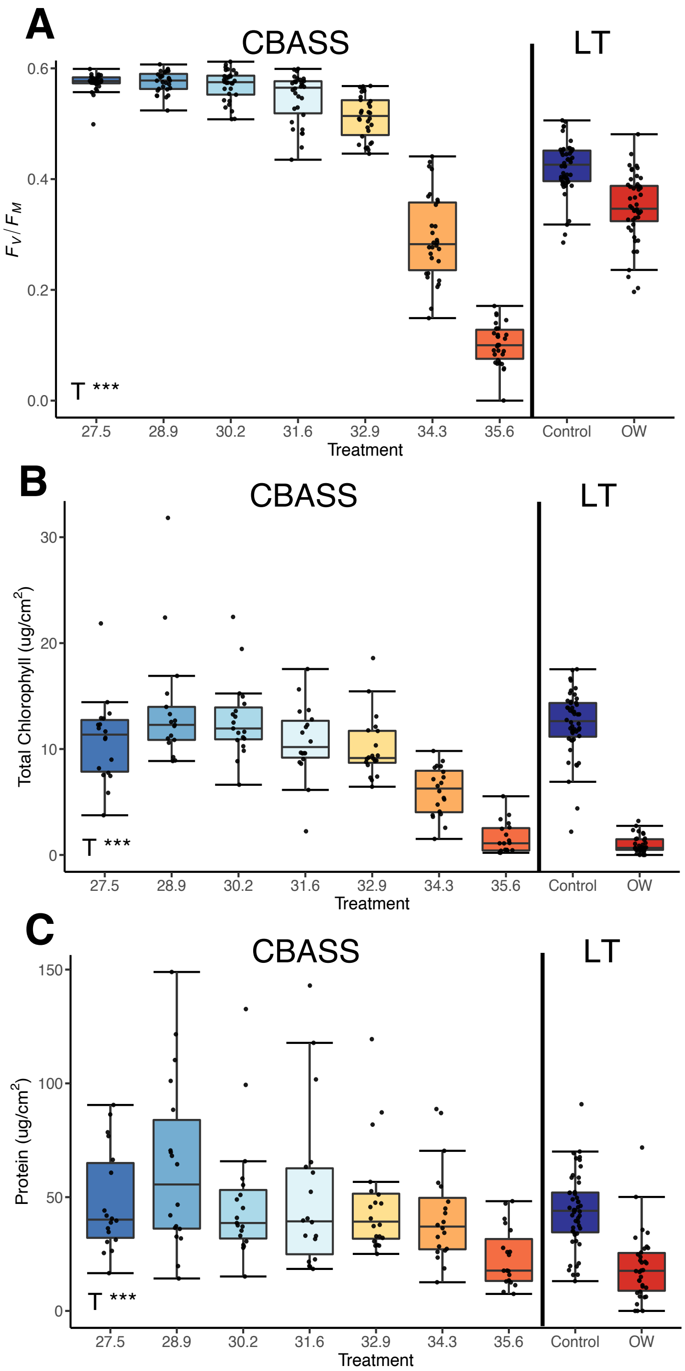

Figure S5. Median boxplots of phenotypic traits of (A) F_v/F_m, (B) total chlorophyll (µg cm^{-2}), and (C) host soluble protein (µg cm^{-2}) measured by treatment in the LT and CBASS experiment. Boxplots for each trait are separated by experiment type, with significant effects treatment (T) reported (*p<0.05, **p<0.01, ***p<0.0001).

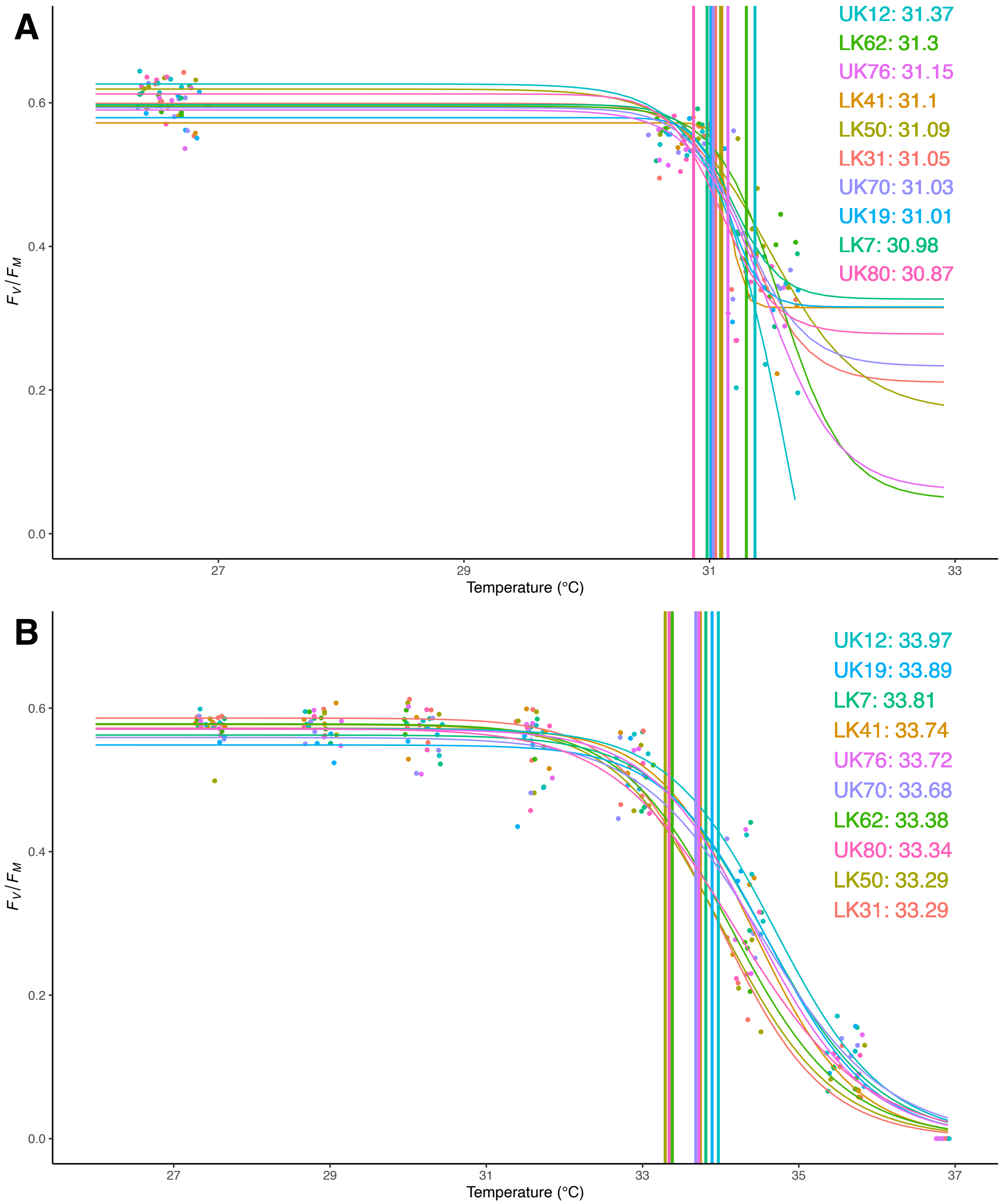

Figure S6. ED25 values of each genotype under (A) LT-OW and (B) CBASS. F_v/F_m values were fit against temperature using a log-logistic dose-response curve. Although non-significant, genotypes are ordered by ED25 values.

Table S7. PERMANOVA pairwise adonis test results of all traits modeled against Experiment (LT and CBASS) treatments.

Pairs	F.Model	R2	p.value	p.adjusted
CBASS-34.3 vs CBASS-30.2	5.79E+02	9.40E-01	0.009	0.315
CBASS-34.3 vs CBASS-35.6	-1.80E+01	-9.50E-01	0.897	1
CBASS-34.3 vs CBASS-28.9	1.70E+02	8.30E-01	0.007	0.245
CBASS-34.3 vs CBASS-31.6	6.40E+01	6.40E-01	0.027	0.945
CBASS-34.3 vs CBASS-27.5	3.26E+03	9.89E-01	0.005	0.175
CBASS-34.3 vs CBASS-32.9	8.21E+03	9.95E-01	0.025	0.875
CBASS-34.3 vs LT-Control	2.67E+02	8.02E-01	0.105	1
CBASS-34.3 vs LT-OW	-3.17E+01	-1.6414178	1	1
**CBASS-30.2 vs CBASS-35.6**	**2.62E+03**	**9.86E-01**	**0.001**	**0.035**
CBASS-30.2 vs CBASS-28.9	1.24E+00	3.51E-02	0.401	1
CBASS-30.2 vs CBASS-31.6	3.92E+01	5.28E-01	0.105	1
CBASS-30.2 vs CBASS-27.5	4.45E+00	1.13E-01	0.24	1
CBASS-30.2 vs CBASS-32.9	-5.81E+00	-0.1864212	0.882	1
CBASS-30.2 vs LT-Control	1.09E+02	6.27E-01	0.322	1
**CBASS-30.2 vs LT-OW**	**1.41E+06**	**1.00E+00**	**0.001**	**0.035**
**CBASS-35.6 vs CBASS-28.9**	**2.48E+05**	**1.00E+00**	**0.001**	**0.035**
**CBASS-35.6 vs CBASS-31.6**	**6.13E+02**	**9.46E-01**	**0.001**	**0.035**
**CBASS-35.6 vs CBASS-27.5**	**1.75E+05**	**1.00E+00**	**0.001**	**0.035**
CBASS-35.6 vs CBASS-32.9	4.41E+02	9.23E-01	0.044	1
CBASS-35.6 vs LT-Control	-1.84E+01	-3.96E-01	0.85	1
**CBASS-35.6 vs LT-OW**	**NaN**	**NaN**	**NA**	**0.035**
CBASS-28.9 vs CBASS-31.6	-6.31E+00	-0.2366371	0.686	1
CBASS-28.9 vs CBASS-27.5	2.14E+00	6.08E-02	0.309	1
CBASS-28.9 vs CBASS-32.9	-6.80E+00	-0.2410131	0.898	1
CBASS-28.9 vs LT-Control	2.90E+01	3.15E-01	0.35	1
**CBASS-28.9 vs LT-OW**	**1.45E+05**	**1.00E+00**	**0.001**	**0.035**
CBASS-31.6 vs CBASS-27.5	7.20E+01	6.79E-01	0.113	1
CBASS-31.6 vs CBASS-32.9	1.03E+03	9.66E-01	0.381	1
CBASS-31.6 vs LT-Control	-1.34E+01	-2.64E-01	0.745	1
**CBASS-31.6 vs LT-OW**	**2.78E+03**	**9.83E-01**	**0.001**	**0.035**
CBASS-27.5 vs CBASS-32.9	4.55E+01	5.58E-01	0.072	1
CBASS-27.5 vs LT-Control	-1.26E+01	-2.44E-01	0.727	1
**CBASS-27.5 vs LT-OW**	**6.25E+04**	**9.99E-01**	**0.001**	**0.035**
CBASS-32.9 vs LT-Control	2.56E+04	9.97E-01	0.305	1
**CBASS-32.9 vs LT-OW**	**8.19E+06**	**1.00E+00**	**0.001**	**0.035**
LT-Control vs LT-OW	1.74E+03	9.57E-01	0.023	0.805

Table S8. Post-hoc Tukey HSD comparisons of Fv/Fm, Total Chlorophyll, and Host Protein values between Treatment (LT-OW [31.5°C] and CBASS 34.3°C) and Region (Upper Keys and Lower Keys)					
**CBASS-34.3°C - LT-OW**					
	**Estimate**	**SE**	**df**	**t.ratio**	**p.value**
A. Fv/Fm					
**Lower Keys**	**-0.255**	**0.0274**	**10.5**	**-9.306**	**<.0001**
**Upper Keys**	**-0.138**	**0.0278**	**11.2**	**-4.957**	**0.0004**
B. Total Chlorophyll					
Lower Keys	-0.0173	0.174	45.1	-0.099	0.9213
**Upper Keys**	**0.5755**	**0.136**	**46.5**	**4.247**	**0.0001**
C. Host Protein					
Lower Keys	0.546	0.735	45.3	0.742	0.4618
**Upper Keys**	**1.286**	**0.566**	**46**	**2.272**	**0.0278**
**Lower Keys - Upper Keys**					
	**Estimate**	**SE**	**df**	**t.ratio**	**p.value**
A. Fv/Fm					
**CBASS 34.3°C**	**-0.0526**	**0.0223**	**68.1**	**-2.362**	**0.021**
**LT-OW**	**0.0642**	**0.0171**	**68.1**	**3.751**	**0.0004**
B. Total Chlorophyll					
**CBASS 34.3°C**	**1.21**	**0.201**	**7.97**	**6.009**	**0.0003**
**LT-OW**	**1.8**	**0.205**	**8.34**	**8.805**	**<.0001**
**C. Host Protein**					
**CBASS 34.3°C**	**1.89**	**0.771**	**9.34**	**2.454**	**0.0356**
**LT-OW**	**2.63**	**0.773**	**9.24**	**3.407**	**0.0075**
